# A pair of duplicated SOC1 genes, *DcSOC1-1* and *DcSOC1-2*, control vernalization requirement in carrot

**DOI:** 10.1186/s12870-026-09529-z

**Published:** 2026-08-08

**Authors:** Josefina Wohlfeiler, Andrés Morales, Claudio R. Galmarini, María Soledad Alessandro, Sofia Carvajal, Douglas Senalik, Shelby Ellison, Alicja Macko-Podgórni, Massimo Iorizzo, Dariusz Grzebelus, Philipp W. Simon, Pablo F. Cavagnaro

**Affiliations:** 1 Instituto Nacional de Tecnología Agropecuaria (INTA) Estación Experimental Agropecuaria La Consulta, La Consulta, Mendoza M5567 Argentina; 2 Consejo Nacional de Investigaciones Científicas y Técnicas (CONICET), Ciudad Autónoma de Buenos Aires, C1425FQB Argentina; 3 Facultad de Ciencias Agrarias, Laboratorio de Biología Molecular, Universidad Nacional de Cuyo, Instituto de Biología Agrícola de Mendoza (IBAM-CONICET), Luján de Cuyo, Mendoza, M5505 Argentina; 4 Catedra de Horticultura y Floricultura, Facultad de Ciencias Agrarias, Universidad Nacional de Cuyo, Lujan de Cuyo, Mendoza, M5528 Argentina; 5 Instituto Nacional de Tecnología Agropecuaria (INTA) Estación Experimental Agropecuaria Mendoza, Lujan de Cuyo, Mendoza, M5507 Argentina; 6 Department of Plant and Agroecosystem Sciences, University of Wisconsin–Madison, Madison, WI 53706 USA; 7 Agricultural Research Service, Vegetable Crops Research Unit, United States Department of Agriculture, Madison, WI United States; 8 Department of Plant Biology and Biotechnology, Faculty of Biotechnology and Horticulture, University of Agriculture in Krakow, Krakow, 31-425 Poland; 9 Plants for Human Health Institute, North Carolina State University, Kannapolis, NC 28081 USA; 10 Department of Horticultural Science, North Carolina State University, Raleigh, NC 27695 USA

**Keywords:** *Daucus carota*, Vernalization, Flowering time, Genetic mapping, Transcriptome, SOC1, Candidate genes

## Abstract

**Background:**

Carrot vernalization requirement (VR), the amount of cold exposure needed for plants to become vernalized, strongly influences geographical adaptation of the crop and many agricultural practices, and this trait defines annual vs. biennial cultivars. Despite its importance, the genetic and molecular mechanisms governing carrot VR remain elusive. Here, we integrated inheritance, linkage mapping, genome wide association studies (GWAS), phylogenetic analysis, and transcriptomics of coding and noncoding mRNA data to dissect the genetic control of carrot VR.

**Results:**

A simply-inherited dominant locus controlling VR, denominated *Vrn1*, was identified and genetically mapped at the distal end of chromosome 2, in a highly-saturated genotyping-by-sequencing (GBS)-derived linkage map. GWAS results revealed a ~ 694 kb region with strong marker-trait associations that overlapped *Vrn1*. In this region, we identified two duplicated SOC1 (SUPPRESSOR OF OVEREXPRESSION OF CONSTANS 1) genes, termed *DcSOC1-1* and *DcSOC1-2*, that were phylogenetically related to SOC1 genes from other species, and were strongly upregulated in vernalized plants relative to non-vernalized plants of annual and biennial carrots. Further, the identification and transcriptome analysis of carrot homologs to Arabidopsis vernalization and flowering genes revealed additional key genes from these pathways exhibiting correlated expression profiles with *DcSOC1-1* and *DcSOC1-2*, including other SOC1, FUL (FRUITFUL) and FLC (FLOWERING LOCUS C) genes. The expression profiles of *DcSOC1-1*, *DcSOC1-2*, and five other vernalization-related genes were validated by RT-qPCR analysis. No differentially expressed ncRNAs directly related to the *Vrn1* candidates were identified.

**Conclusions:**

Overall, this study provides strong linkage-mapping and functional evidences for the candidacy of *DcSOC1-1* and *DcSOC1-2* as the primary regulators of VR in carrot.

**Supplementary Information:**

The online version contains supplementary material available at https://doi.org/10.1186/s12870-026-09529-z.

## Introduction

Vernalization is the process by which a prolonged yet variable period of exposure to low temperatures, which length is genetically-conditioned (i.e., it depends on the species and the genotype within a species) and influenced by environmental factors, renders plants competent to flower [1]. Vernalization plays a central role in the plant’s life cycle, as it conditions the timing of flowering, aligning this developmental stage with important environmental and ecological factors, such as temperature, pollinator activities, and water availability. Evolutionarily, this system is believed to confer adaptive advantages, particularly in species from temperate regions, as it prevents flowering from occurring in winter, postponing the reproductive phase of the plant to the more favorable conditions of spring [2]. Ecologically, this trait strongly influences the adaptation range of some species across different environments, shaping their geographical distribution around the globe; e.g., reducing the requirement for vernalization can facilitate adaptation to habitats that require fast life cycling; for instance, in regions with severe summer aridity; whereas increasing vernalization may be advantageous in higher latitudes [3]. From an agricultural production point of view, vernalization requirement, and thereby flowering time, can affect harvest yield and quality. Therefore, developing varieties with stable flowering time is a major objective for crop breeders, as this facilitates a number of agricultural practices, enabling sustainable yearly productions [4]. Conversely, the possibility of modifying flowering time by means of crop improvement has allowed the adaptation of new varieties to different environmental and/or growing conditions [5].

The genetic regulation of vernalization and the transition to flowering has been extensively studied in the model plant *Arabidopsis thaliana* (hereafter referred to as Arabidopsis). In this species, vernalization and flowering time are controlled by a complex network of flowering-promoting and repressing genes that respond to diverse environmental stimuli, predominantly temperature and photoperiod, and age-related developmental signals (e.g., hormone and carbohydrate levels) [6, 7]. Under this system, the floral repressor gene FLOWERING LOCUS C (FLC), which encodes a MADS domain protein, plays a central role in the vernalization mechanism. FLC, which is highly expressed in vegetative meristems, is epigenetically repressed when plants are exposed to low temperatures for prolonged periods of time, thereby allowing the upregulation of genes that promote flowering. The downregulation of *FLC* by cold is quantitative (i.e., greater accumulation of hours of cold exposure leads to stronger repression of *FLC* activity and earlier flowering) and, once the vernalized state has reached the meristem, it is stable during subsequent mitotic divisions, even after the cold exposure has ended. The cold-induced repression of *FLC* enables the upregulation of key flowering-promoting genes, such as SUPPRESSOR OF OVEREXPRESSION OF CONSTANS 1 (SOC1), FLOWERING LOCUS T (FT), and FLOWERING LOCUS D (FD), among others, that initiate the shift from vegetative to reproductive states in the apical meristem [8]. This mechanism, extensively characterized in Arabidopsis, has been shown to be partially conserved across diverse plant species [9–13], and provides a framework for studying vernalization regulation in other taxa.

In order to flower, carrot plants first need to overcome juvenility, which ends when the plant holds ~ 8–12 leaves and the tap root diameter is 4–8 mm [14–16]. After juvenility, carrot plants are sensitive to the cold stimulus and, after prolonged cold exposure, they become vernalized, after which -and coincidently with the occurrence of warmer temperatures and longer days- they can flower. Carrot vernalization requirement, defined as the amount of cold exposure needed for vernalization, is a genotype-dependent trait used to classify cultivars, typically, as annual (or ‘early flowering’) or biennial (also called ‘late flowering’). A recent study that phenotyped a large germplasm collection based on flowering habit identified sub-categories -based on the timing (e.g., early annuals vs. late annuals) and uniformity of flowering habit within an accession- for the annual and biennial classes [17]. Their results agree with those reported earlier demonstrating variation for VR within annual and biennial accessions [18, 19]. Biennial cultivars require ~ 11–12 weeks at 5 ˚C to complete vernalization and flower [14, 20] whereas annual materials require shorter periods of cold exposure, typically in the range of 5–30 days [16, 21, 22]; and some extreme cases of no cold requirement have been reported in wild carrot accessions [22, 23]. Altogether, these data suggest a broad gradient for VR in the carrot germplasm.

In carrot, VR is an agriculturally important trait with direct implications for root and seed production. If vernalization is completed too fast, relative to root growth, the plant undergoes premature and rapid elongation of the flower stalk, a process known as “bolting”. This is detrimental for commercial root production, as the tap root xylem quickly lignifies, becoming fibrous and unpalatable, which results in a complete loss of commercial value [24, 25]. For seed production, VR strongly influences the dates of flowering, seed set, and harvest; thus, intra-cultivar uniformity for VR is highly desirable, and a major objective in carrot breeding programs. From a production point of view, the VR of a carrot cultivar defines its sowing and harvest dates, based on whether the production purpose is seed or roots, and depending also on the climatic conditions where the crop will be grown.

Little is known about the genetic basis of carrot VR. An initial study by Alessandro and Galmarini [26] examined VR inheritance in carrot segregating populations (F_2_s and BCs) derived from a cross between an annual and a biennial parent, reporting segregation ratios consistent with a monogenic trait, with annual habit being dominant over biennial. Later, this simply-inherited locus, termed *Vrn1*, was genetically mapped to chromosome (Chr.) 2 in an amplified fragment length polymorphism (AFLP)-based linkage map that also included a few simple sequence repeat (SSR) markers, exhibiting tightly linked markers to the trait, spanning a 1.2 cM map region [27]. Considering that 1 cM of the carrot linkage map is equivalent to ~ 0.75 Mbp of the genome sequence [28], the genomic region containing *Vrn1* theoretically spans ~ 0.90 Mbp. Due to the difficulty of aligning non sequence-based AFLP markers to the carrot genome sequence, defining a precise genomic position of *Vrn1* has not been possible to date. In this context, saturating the *Vrn1* region with sequence-based markers, along with expression analysis of the genes in this region in vernalized and non-vernalized plants, would facilitate the identification of candidate genes for *Vrn1*. Very recently, a SOC1 gene located at the end of Chr. 2, which expression promoted early flowering, was proposed as a candidate for *Vrn1* [29]. However, in this study, the authors did not perform genetic mapping or association analysis for VR, and they used a genetic background that is unrelated to the background in which *Vrn1* was originally described and mapped [26, 27].

A previous study compared the flowering behavior and transcriptomes of a cultivated biennial carrot (cv. ‘Amsterdam Forcing’) and a related wild species (*Daucus carota* ssp. *carota* ‘Sognzi’) with annual flowering habit, grown under minimally-vernalizing (14 days at 7 °C) and non-vernalizing conditions [23]. They reported differential gene expression between the annual wild species and the biennial cultivated carrot in 45 flowering-time related genes, including homologues of several photoperiod- and vernalization-response genes. It must be noted that the vernalization conditions used in this study were not sufficient for vernalizing a typical biennial carrot, which usually requires 9–12 weeks at ~ 5 °C [14, 18–20] and therefore their results likely reflect differences between a non-vernalized biennial carrot and a vernalized early-flowering wild carrot. Also, the wild species used was able to flower without cold exposure, showing flowering rates from 40% (under short day conditions) to 100% (under long days), suggesting that it does not require vernalization to induce flowering. Such behavior differs from that of most annual carrot cultivars, which typically require 5–30 days of vernalizing temperatures to induce flowering [16, 18, 21]. Thus, the experimental conditions and plant materials used in the study of Ou et al. [23] do not allow direct extrapolation of their data to comparisons of typical annual vs. biennial cultivated carrots under vernalized and non-vernalized states. Lastly, no candidate genes for VR were reported in that study. Despite these limitations, their results revealed, for the first time, the presence of active flowering-time related genes in this species.

Non-coding RNAs (ncRNAs) are a class of RNAs that do not get translated to protein but can influence the expression of coding genes by different mechanisms [30]. Long non-coding RNAs (lncRNA), typically exceeding 200 nucleotides in length, represent an abundant and functionally relevant subclass of ncRNAs in plants. They can regulate gene expression at various levels by interacting with DNA, RNA, and proteins, thereby influencing the structure and function of chromatin, the transcription of nearby and distant genes, and processes associated with RNA splicing, stability and translation [31]. Antisense transcripts are a subclass of lncRNAs that are transcribed from the opposite strand of the sense transcript of protein-coding and noncoding genes [32]. Antisense transcripts can negatively influence the initiation of the sense transcription by affecting DNA methylation, typically resulting in prolonged suppression of gene expression [33]. An example of such mechanism is *COOLAIR*, a set of antisense transcripts involved in the transcriptional silencing of *FLC* during vernalization, thereby promoting flowering in Arabidopsis [34, 35]. It is possible that a similar regulatory system may exist in carrot. The identification of lncRNAs associated with carrot vernalization has not been reported to date.

The objectives of the present work were to (i) construct a highly-saturated linkage map harboring *Vrn1* using sequence-based ‘genotyping-by-sequencing’ (GBS) markers; (ii) identify candidate genes for *Vrn1* by means of genetic mapping, phylogenetic analysis, and comparative transcriptome analysis of non-vernalized vs. vernalized plants of annual and biennial carrots; (iii) identify carrot genes homologous to Arabidopsis genes involved in vernalization and flowering pathways and analyze their expression profiles -in transcriptome data- across different carrot physiological stages reflecting non-vernalized and vernalized statuses, and investigate their relationship with the *Vrn1* gene candidates; and (iv) validate by RT-qPCR analysis a key subset of genes that regulate *Vrn1.* Through this process, we identified two SOC1 genes, termed *DcSOC1-1* and *DcSOC1-2*, completely linked to the VR locus *Vrn1* and differentially expressed in comparisons of vernalized vs. non-vernalized plants, with expression patterns compatible with their annotated function and correlated with those of other vernalization/flowering genes. Overall, this study provides strong linkage-mapping and functional evidences for the candidacy of *DcSOC1-1* and *DcSOC1-2* as the primary regulators of vernalization requirement in carrot.

## Materials and methods

### Plant materials

Inheritance of vernalization requirement was studied in an F_2_ population derived from a cross between B5, a biennial male-sterile breeding line from the carrot breeding program at INTA La Consulta (Mendoza, Argentina), and Criolla INTA, an annual fertile open-pollinated cultivar from Argentina. To obtain F_1_ progenies, individual B5 plants were crossed with Criolla INTA using isolation cages, and flies were periodically supplied to facilitate cross-pollination. A single fertile F_1_ plant was self-pollinated to produce an F_2_ population, which was grown at the experimental station of INTA La Consulta, Mendoza, Argentina. This F_2_ population (*N* = 1,000) segregated for VR and was used for phenotyping and inheritance analysis. A subset of the population (*N* = 286) was used for the construction of a linkage map containing the phenotypic trait for VR.

### Phenotyping and segregation analysis

The F_2_ population was sown on May 5 of 2016, and the plants were phenotyped on September 5 of the same year for annual or biennial flowering habits. These sowing and phenotyping dates were selected based on previous studies showing to be ideal for discriminating between annual and biennial habits, under the climatic conditions at La Consulta (33°42´S, 69°04´W), Argentina [26]. A total of 1,000 individuals were phenotyped for annual vs. biennial flowering habits, evaluating whether the first floral stem internode had elongated or not, and scored as a binary trait (i.e., presence or absence of flowering) as previously described [26]. Briefly, once per week the plants were evaluated, and they were scored as being ‘vegetative’ until the first floral internode elongated. This stage was soon followed by the appearance of floral primordia, which then progressed to the full development of the primary umbel (Fig. S1). The plants that were not vernalized (i.e., biennial plants) remained in the vegetative stage. The F_2_ population was analyzed using the chi square (χ^2^) method to test whether the proportion of annual and biennial plants fit a 3:1 (annual : biennial) ratio, expected for a single dominant gene conditioning the annual habit, as reported earlier in this same genetic background [26]. The χ^2^ test was performed using R [36].

### Genotyping by sequencing and marker-trait association analysis

Total genomic DNA was isolated from young leaves of individual plants of the F_2_ population a protocol described earlier [37] with modifications for carrot leaves [38]. DNA samples were quantified using the PicoGreen assay (Life Technologies, Grand Island, NY) and they were sent to the Biotechnology Centre of the University of Wisconsin-Madison (Madison, WI, USA) for preparation of genotyping-by-sequencing (GBS) libraries and DNA sequencing. GBS was performed as described by Elshire et al. [39] and Arbizu et al. [40] for carrot. Briefly, DNA was digested with ApeKI methylation-sensitive restriction enzyme, and then samples were barcoded and pooled. Sequencing was carried out on an Illumina NovaSeq 6000 apparatus, using paired-end 150 nt reads and v3 ‘sequencing by synthesis’ reagents (Illumina, San Diego, CA). The data were analyzed using the TASSEL-GBS pipeline version 5.2.39 [41]. Briefly, samples were demultiplexed by matching the barcodes used for each sample and the restriction enzyme site in each individual read, and discarding ambiguous reads without matches. The tags were then exported and aligned to the carrot reference genome v2.0 (GenBank accession LNRQ01000000.1; [28]) with the Burrows–Wheeler Aligner (BWA) software version 0.7.17-r1188. Single nucleotide polymorphisms (SNPs) were identified from the aligned tags by TASSEL. SNPs were then scored according to the coverage, depth, and genotypic statistics for a given set of taxa. The resulting ‘vcf’ file (435,554 loci) was filtered using vcftools version 0.1.16 [42] removing sites without any variant. A total of 152,971 loci were retained. Then, different filters were applied using a step-wise method, as described in Table S1, resulting -at the end of the process- in the retention of 14,675 SNP markers that fulfilled the following criteria: < 1% missing data per locus, > 30% minimum minor allele frequency, and > 10% minimum heterozygous proportion. Associations between markers and phenotypes were carried out using a fixed effects linear model implemented in the ‘Generalized Linear Models’ (GLM) function of TASSEL [41].

To assess the distribution of p-values obtained from the analysis, a quantile-quantile (q-q) plot was generated using the qqman package in R. P-values from 14,675 markers were transformed to the -log10 scale to enhance visualization of significance levels. Expected quantiles were computed using R ppoints function, assuming a uniform distribution under the null hypothesis. The q-q plot was then constructed by plotting the observed and expected -log10(p)-values using the R ggplot2 package (version 3.6.3).

### Genetic map construction

The R/qtl package in R (version 3.6.3) [43] was used for further filtering markers from the GBS data. Markers with more than 10% of missing data were excluded from the analysis, remaining 14,418 SNP markers. Duplicate markers, defined as markers with identical genotypes, were also eliminated, remaining 10,387 SNPs. Markers with significant distortion from the expected 1:2:1 ratio for codominant markers in an F_2_ population, as indicated by χ^2^ tests (*p* < 0.05), were also excluded, leaving 9,415 markers. Lastly, after eliminating redundant markers (i.e., only one marker per recombination event was retained), a total of 1,374 non-redundant SNPs were used for the construction of the linkage map using JoinMap 4.0 [44]. Linkage groups were obtained at a LOD > 10. Regression mapping algorithm and Haldane’s mapping function were used to calculate genetic distances between marker loci. The goodness-of-fit jump was set to 6, and the rest of the parameters used the default settings in JoinMap 4.0.

### Phylogenetic analysis

All analyses were conducted based on the carrot reference genome v2.0 (GenBank accession LNRQ01000000.1; [28]) annotation GCA_001625215.1 (gene symbols DCAR_XXXXXX), supplemented with genes annotated exclusively in NCBI GCF_001625215.1 (gene symbols LOCXXXXXXXXX). Phylogenetic relationships among two carrot SOC1-like genes located in the *Vrn1* region (LOC108209100 and LOC108209496) and MADS-box genes from other species with known regulatory function in different biochemical pathways and physiological processes were investigated. Phylogenetic trees were constructed with the neighbor-joining method, Poisson model, and 1000 bootstrap replicates as parameters in MEGA X software. Also, phylogenetic analysis using these same parameters was performed with DCAR_008644, a putative carrot ‘Bcl-2-associated athanogene’ (BAG) gene and members of the BAG gene family from other species with known functions. Accession numbers and gene names for the protein sequences used in these analyses are presented in Tables S2 and S3.

### Growing conditions and temperature treatments of plants used for transcriptome analysis

In order to simulate natural vernalizing conditions, an experiment under controlled temperature and light conditions was carried out in growth chambers, as follows. Seeds of the annual carrot cultivar Criolla INTA and the biennial line B5 were sown in pots (10 cm diameter × 18 cm height) containing fertile soil (14% organic material, pH 6.8, and a carbon-to-nitrogen ratio of 15). The plants were grown at a mean temperature of 25 ˚C, long-day photoperiod [16 h of light (average light intensity was 169 µmol m^− 2^ s^− 1^) and 8 h of darkness]. During this period, a subset of juvenile plants from each carrot line were sampled for RNA isolation and sequencing (described below). For the remaining plants, when they had 8–12 leaves, and juvenility was assumed to have ended [14, 16], they were transferred to another chamber with vernalizing conditions, with a mean temperature of 5 °C and a short-day photoperiod (8 h of light and 16 h of darkness), for 40 or 80 days. A subset of the plants of each carrot line remained in the growth chamber with 25 ˚C and long-day regime, as non-vernalized controls. When the cold treatment finished, after 40 or 80 days, respectively, plants from both carrot lines were sampled for RNA isolation and sequencing. A subset of the pots from the 80-days cold treatment were transferred back to the growth chamber with non-vernalizing conditions (25 ˚C, long days) until flowering (for ~ 25 days), and the inflorescence of the flowering plants were sampled for RNA isolation and sequencing. During the entire experiment, the plants were watered twice a week, and the pots were distributed in the chamber using a completely randomized design to eliminate any location effect. A scheme of the experimental design described above is presented in Fig. S2.

### RNA sequencing and transcriptome analyses

#### Tissue sampling and RNA isolation

The apical meristems from 15 to 20 plants of each carrot line, B5 and Criolla INTA, were dissected and combined, in pools of five meristems each, at each of five time-points, representing five different physiological stages, as follows: (1) juvenility (the plants had not yet developed the 8–12 leaves, indicative of termination of the juvenile phase) (Juv); (2) 40 days of cold treatment (40d); (3) 80 days of cold treatment (80d); (4) after flowering (Flw); and (5) control plants (Ctl) that were grown under non-vernalizing conditions for 80 additional days after they had ended the juvenile stage, equivalent in chronological age to stage 3 (Fig. S2). Thus, this sampling method yielded 3–4 biological replicates, being each replicate a pool of five meristems derived from five individual plants, for each of the ‘cultivar × time-point’ combinations, with only one exception containing two replicates, namely stage 4 (flowering) for B5 (Table S4). The meristems were dissected using a scalpel, then they were flash-frozen in liquid nitrogen, and stored at -80 °C until total RNA isolation was performed. Noteworthy, B5 and Criolla INTA are highly stable for the expression of VR, as demonstrated in several previous studies [18, 26, 27, 45].

Total RNA was extracted using the Promega SV Total RNA Isolation System kit (Promega Corporation, Madison, WI, USA) following the protocol provided by the supplier. DNA was removed with a DNase I solution provided with the kit. RNA quantification was performed in a DeNovix DS-11 spectrophotometer, and quality control was carried out using an Agilent 2100 Bioanalyzer RNA NanoChip. RNA samples were sent to the University of Wisconsin-Madison Biotechnology Center for library preparation and RNA sequencing.

#### Library construction, RNA sequencing and processing

Paired-end libraries with 151-nt insert size were prepared for each sample using the TruSeq Stranded Total RNA (Plant, rRNA reduction) library, and they were sequenced on Illumina NovaSeq 6000 lanes using 2 × 150 nt reads. After quality control with FastQC (http://www.bioinformatics.babraham.ac.uk/projects/fastqc/), reads were filtered with Trimmomatic version 0.39 [46] using the following parameters: phred33 ILLUMINACLIP: adapter.fna:4:30:10, SLIDINGWINDOW:4:15 LEADING:20 TRAILING:20 MINLEN:36. In this process, adapter sequences were removed from the reads and low-quality bases were trimmed, and the quality of the remaining sequences was checked again with FastQC. For removing reads corresponding to residual ribosomal RNA, trimmed reads were mapped to the rRNA reference from carrot using SortMeRNA with ´--ref silva-bac-16s-id90.fasta --ref silva-bac-23s-id98.fasta --ref silva-arc-16s-id95.fasta --ref silva-arc-23s-id98.fasta --ref silva-euk-18s-id95.fasta --ref silva-euk-28s-id98.fasta --ref rfam-5s-database-id98.fasta --ref rfam-5.8s-database-id98.fasta --num_alignments 1 --out2 --blast --fastx –v´ as parameters. Table S4 presents further information on the RNA-Seq libraries and resulting reads.

#### New transcripts assembly and lncRNA identification

Clean filtered reads were aligned to the *Daucus carota* genome sequence [28] using the STAR aligner with ´--outSAMtype BAM Unsorted --quantMode TranscriptomeSAM GeneConts --quantTranscriptomeBan Singleend --outReadsUnmapped Fastx --outFilterScoreMinOverLread 0.3 --outFilterMatchNminOverLread 0.3´ as parameters. The aligned reads were assembled using Stringtie version 2.2.1 [47] and new transcripts were extracted and annotated using Gffcompare v0.12.6 and Infernal v1.1.2.

#### Differential expression analysis

Strand-specific read counts of coding and non-coding genes was performed using the carrot genome annotation GCA_001625215.1 (gene symbols DCAR_XXXXXX), supplemented with genes annotated exclusively in NCBI GCF_001625215.1 (gene symbols LOCXXXXXXXXX) for each of the 31 aligned BAM files by means of the featureCounts function included in the Rsubread package [48]. Differentially expressed genes (DEGs) were identified in all the pairwise comparisons with edgeR (version 3.28.1) and limma (version 3.42.2) packages [49] in R (version 3.6.3). Reads were filtered to retain only transcripts with more than ten reads per million mapped reads in at least two libraries, and the normalization factor was calculated for each sample with the trimmed mean of M-values (TMM) proposed by Robinson and Oshlack [50]. DEGs were declared significant when having a log-fold-change ≥ 1 and a false discovery rate (FDR)-adjusted p-value < 0.05, as calculated from empirical Bayes moderated t-statistic tests.

### Gene expression analysis by RT-qPCR

Transcript levels of seven selected vernalization/flowering related genes that were differentially expressed based on RNA-Seq data were validated by RT-qPCR analysis in the meristems of cold-treated plants and non-vernalized plants of B5 and Criolla INTA at four physiological and developmental stages (Juv, Ctl, 40d, and 80d). To this end, independent plants from the same experiment used for transcriptome analysis were sampled. Sampling of meristems, and RNA isolation and quantification were performed as described in previous sections. cDNA was synthesized from 1 µg of RNA by Invitrogen™ SuperScript™ III First-Strand Synthesis System (Thermo Fisher Scientific, MA) and diluted 25-fold for RT-qPCR analysis. RT-qPCR reactions were carried out using the SYBR™ Green PCR Master Mix (Thermofisher Scientific) in a StepOne™ Real-Time PCR system (Applied Biosystems, USA) using the following amplification conditions: a first step of enzymatic activation at 95 °C for 10 min, followed by 40 cycles of 95 °C for 15 s (denaturation) and 60 °C for 1 min (annealing and elongation). Analysis of melting curves was performed to verify the amplification specificity. To this end, the dissociation curve protocol started immediately after amplification and consisted of 1 min at 60 °C followed by steps of 80 –10 s with temperature increments of 0.3 °C at each step, until reaching 95 °C. Reactions were run in triplicates. Relative quantification of gene expression was performed according to Pfaffl (2001) with the modification proposed by Ruijter et al. [51], which takes into account the amplification reaction efficiencies [52] using the ‘Differential Gene Expression Analysis’ package from fgStatistics Software version 2012 [53]. The actin gene was used as an internal control to normalize the variability in expression levels [54]. By this means we obtained the fold changes in gene expression relative to the internal control gene (actin), and the data were expressed as the ratio ∆Cq-target gene/∆Cq-actin. Information on the primers used for RT-qPCR analysis is presented in Table S5.

### Search of candidate genes conditioning *Vrn1*

To identify putative candidate genes conditioning vernalization requirement, the sequences surrounding the SNP markers flanking the *Vrn1* map region were aligned to the carrot genome assembly DCARv2 [28] and the resulting sequence between the two markers was used to search for candidate genes. To this end, gene predictions from DCARv2 (GCA_001625215.1), and RefSeq (NCBI Reference Sequences; GCF_001625215.1) gene models not overlapping with any gene predicted in DCARv2, were extracted and used to search for homologous sequences with functional annotations from other species in the protein database Swiss-Prot, using ‘blastp’ as search tool. Downstream analyses were performed (as described in previous sections) to find key genes conditioning *Vrn1*, requiring the following criteria for declaring *Vrn1* candidates: (i) localization of the gene within the *Vrn1* genomic region, as defined above; (ii) functional annotation associated with the vernalization pathway, and/or phylogenetic relatedness to genes from other species known to be involved in the vernalization pathway; (iii) differential expression in transcriptome comparisons of vernalized vs. non-vernalized samples within each carrot line (B5 and Criolla), and in agreement with the putative function of the gene; and (iv) validation of transcriptome results by RT-qPCR analysis. The genes fulfilling these four criteria were considered major candidates for *Vrn1*.

### Genome structure analysis of the *Vrn1* region

A comparison of the genomic region significantly associated with *Vrn1* between *Daucus carota* DH1.v2 ([28]; GCA_001625215.1) and DH1.v3 ([54]; GCF_001625215.2), and the identification of putatively duplicated regions, was performed using blastn from BLAST 2.9.0+ [55] with the parameters -dust no -soft_masking false. The results were visualized with genoPlotR v0.8.11 [56], combined with gene (NCBI GCF_001625215.2-RS_2024_03) and repeat annotations. Repeat annotation was done with EarlGrey v4.4.0 [57], using the previously published carrot transposable element (TE) database [54].

### Expression analysis of other genes involved in carrot vernalization and flowering

Protein sequences from 107 Arabidopsis genes involved in the vernalization and flower development pathways, known to influence flowering time in this species, were retrieved from the FLOR-ID database [58] (Table S6), and the homologous carrot genes were identified by performing blastp searches from BLAST 2.9.0+ [55] using the Arabidopsis sequences as queries, against carrot proteins (Phytozome v12: Dcarota_388_v2.0.protein.fa); and target sequences with an e-value < 1e-10 were considered. Carrot proteins showing ≥ 40% identity and ≥ 50% query coverage were retained and subsequently used as queries in a second round of blastp search against the Arabidopsis protein database, with -max_target_seqs 1 applied to retrieve the closest Arabidopsis homolog. The resulting set of carrot genes homologous to Arabidopsis vernalization and flower development-related genes (Table S7) were analyzed for differential expression in the carrot transcriptome data.

## Results

### Inheritance and genetic mapping of the carrot vernalization requirement locus *Vrn1*

Phenotyping of 1000 F_2_ individuals for annual or biennial flowering habits revealed a 3:1 annual : biennial ratio (χ²=0.01, *p* = 0.99), suggesting a single dominant gene for the genetic control of the annual vs. biennial habits. Because we used the same genetic background as previously reported for *Vrn1* [26, 27], and the annual/biennial flowering habits segregated in the same fashion as reported in these previous studies, the same term, *Vrn1*, was used to designate the locus conditioning vernalization requirement in the present work.

**Fig. 1:**
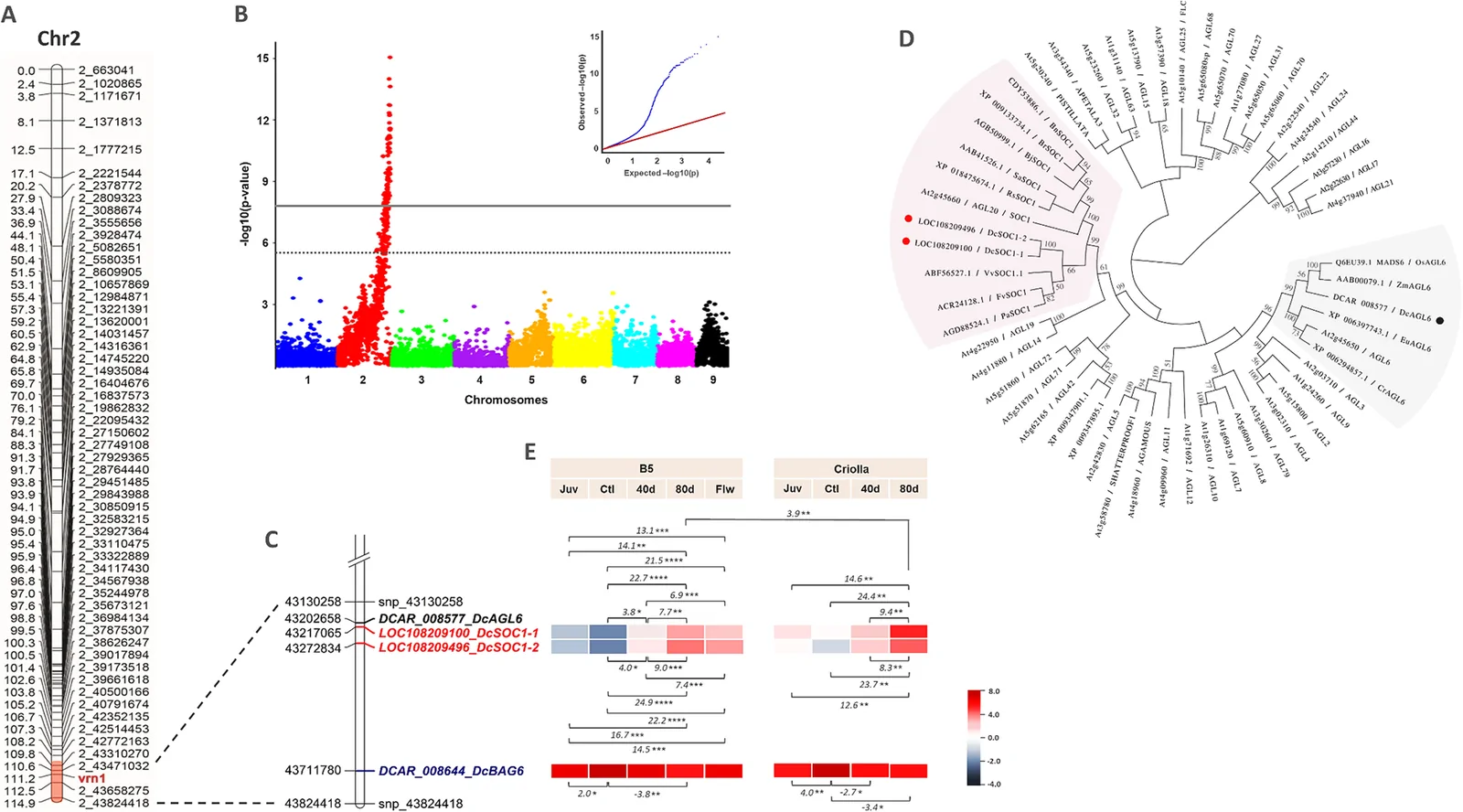
Identification and analysis of candidate genes controlling carrot vernalization requirement (*Vrn1*). **A** Genetic linkage map of chromosome 2 including *Vrn1* (in red font). Map positions in centimorgans (cM) are indicated on the left of each SNP marker (designated by the chromosome number followed by its genome coordinates in nucleotides). The region with strongest association with vernalization requirement, as evaluated by association mapping, which was used to search for candidate genes, is highlighted in red. **B** Manhattan plot summarizing association mapping results for vernalization requirement. The horizontal dotted and solid lines indicate significance thresholds based on the Bonferroni and false discovery rate (FDR) methods for *p* = 0.05, respectively. The image in the upper right corner shows q-q plot for 14,675 markers with expected and observed quantiles, expressed as -log10(p) values, denoted in red and blue lines, respectively. **C** Magnified image corresponds to the physical map, in nucleotides, of the *Vrn1* region, and includes four genes potentially involved in the vernalization and flowering pathways. Genes with significant differential expression for at least one of the transcriptome comparisons are color-coded in red, for SOC1-like genes (*DcSOC1-1*, *DcSOC1-2*), and blue, for *DcBAG6*. **D** Phylogenetic relationships among the three carrot MADS-box genes identified in the *Vrn1* region and MADS-box genes with known function from other species. Clusters comprising SOC1 and AGL6 sequences are indicated in pink and gray colors. Red and black dots indicate carrot SOC1-like and AGL6 genes, respectively. Bootstrap values ≥ 50 are indicated. More information on the sequences and species used for the analysis is presented in Table S2. **E** Heat map depicting expression patterns (RNA-Seq) of *DcSOC1-1*, *DcSOC1-2*, and *DcBAG6* across five developmental/physiological stages, including two non-vernalized [juvenile (Juv) and control (Ctl)] and three potentially vernalized stages [after 40 days of cold (40d), after 80 days of cold (80d), and after flowering (Flw)] in B5 and Criolla. A diagram depicting the experimental design and growing conditions is presented in Fig. S2. Criolla lacked the ‘Flw’ stage. Each cell of the heat map represents the log expression, calculated as the mean ‘transcripts per million’ (TPM) value from the expression profiles of 3–4 replicates per sample set. Fold changes and p-values [< 0.05 (*), < 0.01 (**), < 0.001(***), and < 0.0001 (****)] are indicated for statistically significant pairwise comparisons of expression levels, calculated as described in materials and methods. Expression data for *DcAGL6* were not included, as this gene was not differentially expressed in all the comparisons examined

A subset of the F_2_ population (*N* = 239), presenting the same phenotypic segregation ratio for the two flowering habits as in the larger population used for inheritance analysis, was used to identify the region harboring *Vrn1*, by means of genetic linkage mapping and marker-trait association analysis by ‘genome wide association studies’ (GWAS). A high-resolution linkage map (mean distance between two contiguous non-redundant markers was 0.96 cM, with a total map distance of 916.7 cM, equivalent to 360,397,739 nt) was constructed. The resulting linkage map is presented in Figs. 1A and S3, and detailed information about the phenotypes and genotypes of each of the F_2_ plants used for mapping, and main characteristics of the obtained linkage groups are presented in Tables S8-S9. Linkage mapping placed *Vrn1* at the end of chromosome 2, at a position of 111.2 cM, with its immediate flanking markers spanning a 1.9 cM region, from 110.6 (for SNP marker 43,471,032) to 112.5 cM (for marker 43,658,275) (Fig. 1A). By aligning these flanking markers to the reference carrot genome sequence [28], it was determined that the region harboring *Vrn1* corresponds to 187,243 nt of the carrot genome sequence. *Vrn1* and its immediate upper and lower flanking markers had high statistical support for their map positions, as indicated by their LOD values of 12.3, 17.6, and 18.1, respectively. Results from the GWAS analysis coincided with those of linkage mapping, and showed overlapping map positions for this phenotypic trait at the distal end of chromosome 2 (Fig. 1A, B). GWAS revealed a region at the end of Chr. 2 with significant and strongest marker-trait associations for 44 SNP markers (mean p-value was 3.3e-10, with a range of 5.0e-09–8.8e-16; and mean -Log10(p) was 11.0, with a range of 8.3–15.1), spanning a 694,160 nt region, from 43,130,258 to 43,824,418 (Fig. 1A-C; Tables S10-S11). No clear differentiation among these 44 markers could be established for their degree of trait-association to define a narrower region for *Vrn1*. This ~ 694 kb region of Chr. 2 was further examined for candidate genes conditioning *Vrn1*.

### Identification and analysis of candidate genes conditioning *Vrn1*

Using the gene prediction from the carrot genome assembly DCARv2 and RefSeq gene models, 109 genes were identified in the *Vrn1* region (Table S12). Among them, four genes had functional annotations directly related to vernalization and/or flowering pathways. These include a cluster of three MADS-box genes identified within a ~ 77 kb region (with coordinates 43,202,658 − 43,279,748), two of which, namely LOC108209100 and LOC108209496, were annotated as SOC1-like genes, and revealed high homology with SOC1 genes from Arabidopsis and other species, at the protein and mRNA levels (percent identity was 67.5–77.6% and 77.6–80.8%, respectively; Table S13). In Arabidopsis, SOC1 is considered a flowering time regulator that plays a central role in the transition to flowering, integrating the vernalization, autonomous, and photoperiod pathways [59]. The other MADS-box gene (DCAR_008577) was homologous to AGAMOUS-LIKE 6 (AGL6) proteins from other species (Table S13). Lastly, the fourth candidate gene found in the *Vrn1* region is a molecular chaperone regulator 6 of the Bcl-2-associated athanogene (BAG) gene family, which was termed *DcBAG6*. In Arabidopsis, AGL6 acts as a floral promoter that negatively regulates FLC and MADS AFFECTING FLOWERING (MAF) genes and positively regulates FT [60], whereas an homolog of *DcBAG6*, namely *atbag6*, has been suggested to act as a flowering repressor [61].

Phylogenetic analysis using the predicted protein sequences from these three carrot MADS-box genes and 53 MADS-box genes with known functions from other species (Table S2) revealed clustering of the two carrot SOC1-like genes with SOC1 genes from Arabidopsis and nine other species (Fig. 1D). The carrot SOC1-like genes were phylogenetically closest, at a protein sequence level, to each other and to SOC1 genes from *Vitis vinifera* (accession number ABF56527.1), *Fragaria vesca* (ACR24128.1), and *Prunus armeniaca* (AGD88524.1). The other carrot MADS-box, DCAR_008577, was clustered separately from the SOC1-like genes, and was included in a clade with five AGL6 genes from other species, being phylogenetically closest to AGL6 genes from *Zea mays* (AAB00079.1) and *Oryza sativa* (Q6EU39.1). These results suggest that LOC108209100 and LOC108209496 may indeed function as SOC1 genes in carrot, and they were termed *DcSOC1-1* and *DcSOC1-2*, respectively, whereas DCAR_008577, a possible carrot AGL6, was termed *DcAGL6*. Additionally, phylogenetic analysis of *DcBAG6* along with 116 protein members of the BAG family from other species with known functions, revealed phylogenetic clustering of *DcBAG6* with 34 BAG6 proteins from other species, being phylogenetically closest to sequences from *Sesamum alatum* (KAK4415886.1) and *Olea europaea* (CAA2977647.1) (Fig. S4). These results suggest that *DcBAG6* may indeed act as a BAG6 chaperone regulator with similar functions as the homologous Arabidopsis gene *atbag6*; i.e., negatively regulating flowering time. Altogether, these four genes identified in the *Vrn1* region (*DcSOC1-1*, *DcSOC1-2*, *DcAGL6*, and *DcBAG6*), with predicted functions associated with vernalization/flowering processes, were considered preliminary candidates of *Vrn1*, and were further analyzed.

Comparative analysis of transcriptomes from the shoot apical meristem of carrot plants at five different developmental and physiological stages, representing non-vernalized plants (NVP) [i.e., Juvenility (Juv) and non-vernalized controls (Ctl)] and cold-treated plants (CTP) [i.e., after 40 (40d) and 80 days of cold treatment (80d), and after flowering (Flw)], in both carrot lines (Criolla INTA and B5), was performed; and the results for the four *Vrn1*-candidate genes are presented in Fig. 1E. Both SOC1 genes, *DcSOC1-1* and *DcSOC1-2*, were differentially expressed among physiological stages in both carrot lines, with their expression patterns fully coinciding with the proposed role of SOC1 genes as flowering integrators that induce flowering. Thus, comparing NVP (Juv, Ctl) with any of the CTP (40d, 80d, Flw) revealed strong upregulation of *DcSOC1-1* and *DcSOC1-2* in the latter group, with upregulation increasing with longer cold exposure, in both carrot lines. Thus, in B5, their expression levels increased 3.8-4.0 fold (*p* < 0.05) after 40 days of cold, and 21.5–24.9 fold (*p* < 0.0001) in 80d and Flw samples, relative to Ctl samples. Similar results were found for comparisons of juvenile vs. cold-treated plants of B5, revealing upregulation of both SOC1 genes in the latter, with increases in expression levels in the range of 13.1–16.7 fold (*p* < 0.001) for 80d and Flw. Comparisons between juvenile plants and plants under the shortest cold treatment (40d) also showed upregulation in the latter, but the increase did not reach statistical significance. For the annual carrot Criolla INTA, comparable results were observed with regards to the expression patterns of SOC1 genes. Thus, comparisons between NVP (Ctl and Juv) and CTP (40d and 80d) revealed strong upregulation of both SOC1 genes in 80d plants, with 12.6–24.4 fold increases in expression levels (*p* < 0.01). As observed for B5, the differences in expression levels between NVP plants and plants grown under the shortest cold treatment (40d) did not reach statistical significance, although the cold-treated samples increased transcript levels of both genes. Also, upregulation of *DcSOC1-1* and *DcSOC1-2* in Criolla INTA increased significantly (*p* < 0.01) with longer cold exposure, as indicated by the 8.3–9.4 fold increase found in SOC1 transcript levels in 80d samples relative to 40d. Interestingly, when comparing CTP between both carrot lines, *DcSOC1-1* appeared significantly upregulated in 80d samples of Criolla INTA relative to the same time-point in B5, exhibiting a 3.9-fold difference in expression level (*p* < 0.05), suggesting a deeper vernalization status in Criolla INTA relative to B5 for the same time point, in agreement with their annual and biennial flowering behavior, respectively. Such differences were not observed for *DcSOC1-2*. Together, these data suggest that *DcSOC1-1*, but not *DcSOC1-2*, is a key gene conditioning annual vs. biennial flowering habits in carrot. Altogether, these results indicate that *DcSOC1-1* and *DcSOC1-2* are strong candidate genes of *Vrn1*, conditioning flowering time and vernalization requirement in carrot. The other MADS-box gene found in the *Vrn1* region, *DcAGL6*, was not differentially expressed among physiological stages of neither carrot lines, suggesting that it is not a *Vrn1* candidate, and therefore it was not further analyzed.

Expression analysis of *DcBAG6*maximum transcripts level in NVP undergoing active vegetative growth, and increased from Juv to Ctl plants, but then showed reduced expression in cold-treated plants (40d, 80d) relative to Ctl plants in both carrot lines (Fig. 1E). Thus, expression levels of *DcBAG6* dropped 3.8 fold (*p* < 0.01) from Ctl to 80d in B5; and 2.7–3.4 fold (*p* < 0.05) from Ctl to 40d and 80d samples in Criolla INTA. These expression patterns, and the fact that the annual carrot (Criolla INTA) revealed a significant reduction in the expression of this gene after 40 and 80 days of cold, in contrast to the reduction observed only after 80 days of cold in the biennial carrot (B5), aligns with both the proposed role of this gene as a flowering inhibitor and the annual and biennial flowering habits of the carrot lines, suggesting that inhibition of flowering is suppressed/reduced earlier in annual carrots than in biennial ones. These data, together with the expression profile of *DcSOC1-1* showing significantly greater expression in 80d samples of Criolla INTA compared to B5, support, at the molecular level, the fact that annual carrots have lower vernalization requirements than biennial carrots.

### Structural analysis of the genomic region harboring *Vrn1* and its candidate genes *DcSOC1-1* and *DcSOC1-2*

**Fig. 2:**
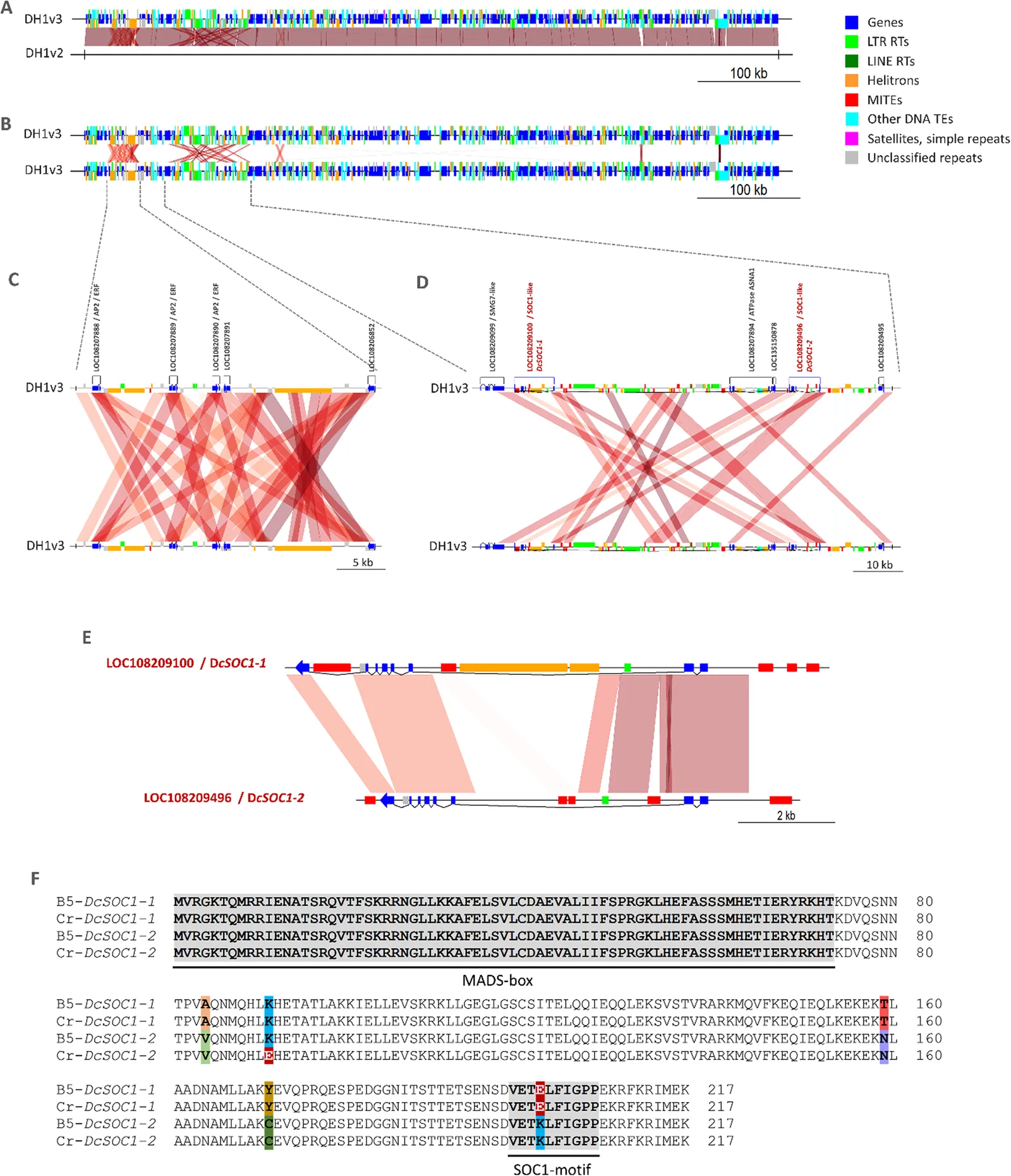
Structural analysis of the genomic region harboring *Vrn1* and its candidate genes *DcSOC1-1* and *DcSOC1-2*. **A** Comparison of the *Vrn1* region in DH1v3 (GCA_001625215.2; chr2: 58343672–59035878) and the respective region in DH1v2 (GCA_001625215.1; chr2: 43130258–43824418). BLAST results with minimum length > 1 kb, maximum e-value < 1e-05, and minimum identity > 70% are shown. **B-D** Self-BLAST analysis of the *Vrn1* region (chr2: 58,343,672 − 59,035,878) showing duplications in the entire 694 kb-region (**B**), in region ‘chr2: 58,368,243 − 58,398,503’ (**C**), and in region ‘chr2: 58,422,766 − 58,506,317’. **E** Comparison of *DcSOC1-1* and *DcSOC1-2.* Blue boxes indicate exons and connecting lines indicate introns. BLAST parameters are as described above. LTR RTs. Long terminal repeat retrotransposons; LINE RTs. Long interspersed nuclear element retrotransposons; MITEs. Miniature inverted–repeat transposable elements. **F** Amino acid sequence alignment of *DcSOC1-1* and *DcSOC1-2* from B5 and Criolla (Cr). The conserved MADS-box domain and SOC1-specific motif are indicated with underlines, and amino acid substitutions are highlighted in color

Structural analysis of the ~ 694 kb region associated with *Vrn1*, harboring *DcSOC1-1* and *DcSOC1-2*, is shown in Fig. 2. The original DH1v2 (GCA_001625215.1) and the improved DH1v3 (GCA_001625215.2) carrot reference genome assemblies did not differ markedly at the *Vrn1* region (Fig. 2A). Two highly duplicated regions were identified within the *Vrn1* region (Fig. 2A-D). The first one comprised ~ 30.2 kb and included three highly-homologous genes annotated as ‘AP2/ERF and B3 domain-containing transcription factors’, two duplicated genes with uncharacterized functions, and various transposable elements (TEs) (Fig. 2C). The second region was larger (~ 83.6 kb) and harbored six genes, including *DcSOC1-1* and *DcSOC1-2*, two genes encoding putative SMG7-like and ATPase ASNA1 proteins, and two genes with unknown functions; along with several transposable elements belonging to different TE families (Fig. 2D). Comparison of the two *SOC1* genes revealed high sequence similarity, particularly in the coding regions, as well as in their 5’-end non-coding and upstream regions (Fig. 2E). However, distinctive features were noticed in the non-coding regions, particularly the presence of a *Helitron* TE in the second intron and a MITE insertion in the last intron of *DcSOC1-1*, which were absent in *DcSOC1-2*, whereas the latter carried an additional MITE insertion the second intron. Also, there are MITE insertion in the upstream (5’-end) region of both genes, with *DcSOC1-1* presenting three MITE insertions and *DcSOC1-2* having only one.

In the reference genome of DH1, *DcSOC1-1* (LOC108209100) has a total length of 8,938 nt, with eight exons and seven introns of varying sizes. *DcSOC1-2* (LOC108209496) is shorter, with a total length of 6,915 nt, also containing eight exons and seven introns. Most of the variation in length between the two genes was due to differences in intron sizes, particularly in introns 2 and 7. The predicted mRNA sequence of *DcSOC1-1* comprises 965 nt while *DcSOC1-2* mRNA is 982-nt-long (Fig. S5). The coding regions (CDS) of both genes have 654 nt, with only 10 nucleotide substitutions (Fig. S6). The predicted protein sequences of both genes comprise 217 amino acids (AA), with an identity of 98%, and contain the characteristic MADS-box domain at the N-terminus and the SOC1-motif at the C-terminus (Fig. S7).

Comparative analysis of the full transcript sequences of *DcSOC1-1* and *DcSOC1-2* from B5 and Criolla INTA, obtained from the RNA-Seq data, revealed a total length of 965 nt for all the transcripts, showing high level of sequence identity (> 98%) with only 17 nucleotide polymorphisms found among the four mRNAs (Fig. S8). Predicted protein sequence alignment revealed that *DcSOC1-1* shared high amino acid sequence identity (> 97%) with *DcSOC1-2* in both carrot accessions, confirming their status as tandem duplicates, and similar identity levels were found for the two SOC1 genes when compared between the accessions (Fig. 2F). All the proteins had 217 amino acids and contained the characteristic MADS-box domain and the SOC1-motif; and comparison of the two SOC1 proteins within each accession evidenced the same amino acid substitutions as in DH1 (Figs. 2F, S7). The latter observation suggests that the duplication of these SOC1 genes precedes the diversification of cultivated carrots.

### General transcriptome analysis and identification of other genes involved in carrot vernalization and flowering

**Fig. 3:**
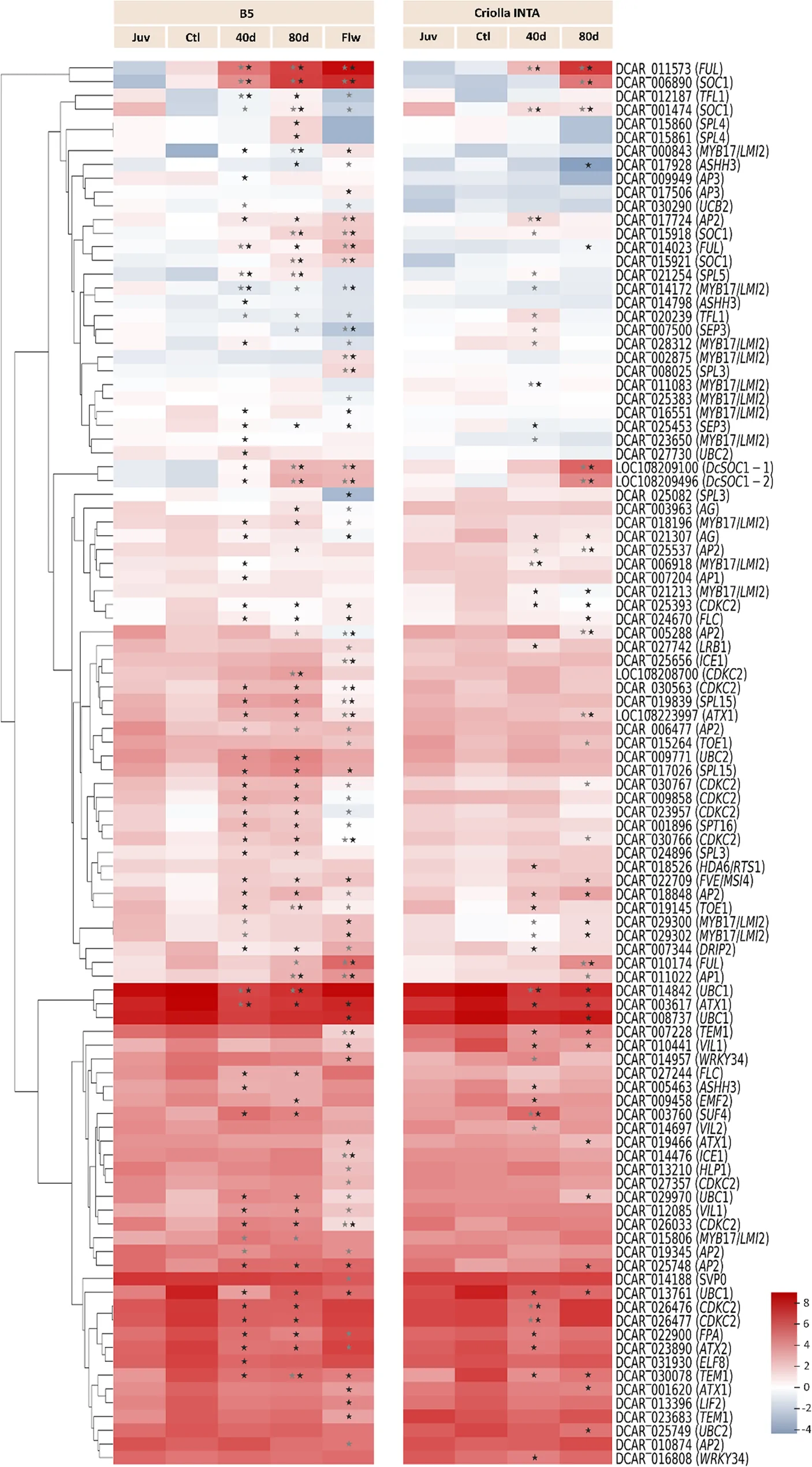
Differential expression of vernalization and flowering development-related genes across physiological and developmental stages in B5 and Criolla INTA. Heatmap based on hierarchical clustering analysis of normalized gene expression data expressed as log counts per million (logCPM). Each column of the heat map represents the mean value from the expression of 3–4 biological replicates. Only genes with statistically differential expression between CTP (40d, 80d, Flw) and non-vernalized (Juv, Ctl) samples are presented (i.e., genes with differential expression only between vernalized or between non-vernalized samples are not presented). Black and gray stars indicate statistically different expression of the vernalized samples relative to the expression in Ctl and Juv samples, respectively. Name abbreviations of the homologous Arabidopsis genes are shown in parenthesis. *AG*: *AGAMOUS*; *AP1*: *APETALA1*; *AP2*: *APETALA2*; *AP3*: *APETALA3*; *ASHH3*: histone-lysine N-methyltransferase ASHH3; *DRIP2*: DREB2A-interacting protein 2; *ATX1*; Arabidopsis trithorax 1; *ATX2*: Arabidopsis trithorax 2; *CDKC2*: Cyclin dependent kinase group C2; *ELF8*: *EARLY FLOWERING 8*; *EMF2*: *EMBRYONIC FLOWER 2*; *FLC*: *FLOWERING LOCUS C*; *FPA*: FPA RNA binding protein; *FUL*: *FRUITFULL*; *FVE*: Flowering locus VE; *HDA6*: HISTONE DEACETYLASE 6; *HLP1*: hnRNP A1-like protein 1; *ICE1*: INDUCER OF CBF EXPRESSION 1; *LIF2*: LHP1-INTERACTING FACTOR 2; *LRB1*: LIGHT-RESPONSE BTB 1; *MYB17/LMI2*: *myb domain protein 17* / *LATE MERISTEM IDENTITY 2*; *SEP3*: *SEPALLATA 3*; *SOC1*: *SUPPRESSOR OF OVEREXPRESSION OF CONTANS 1*; *SPL15*: *SQUAMOSA PROMOTER BINDING PROTEIN-LIKE 15*; *SPL3*: *SQUAMOSA PROMOTER BINDING PROTEIN-LIKE 3*; *SPL4*: *SQUAMOSA PROMOTER BINDING PROTEIN-LIKE 4*; *SPL5*: *SQUAMOSA PROMOTER BINDING PROTEIN-LIKE 5*; *SPT16*: Suppressor of Ty 16; *SUF4*: *Suppressor of FRIGIDA 4*; *SVP*: *SHORT VEGETATIVE PHASE*; *TEM1/EDF1*: *TEMPRANILLO 1 / ETHYLENE RESPONSE DNA BINDING FACTOR 1*; *TOE1*: *TARGET OF EARLY ACTIVATION TAGGED (EAT) 1*; *UBC1*: *UBIQUITIN CARRIER PROTEIN 1*; *UBC2*: *UBIQUITIN CARRIER PROTEIN 2*; *VIL1*: *VIN3-LIKE 1*; *VIL2*: *VIN3-LIKE 2*; *WRKY34*: *WRKY DNA-BINDING PROTEIN 34*

**Fig. 4:**
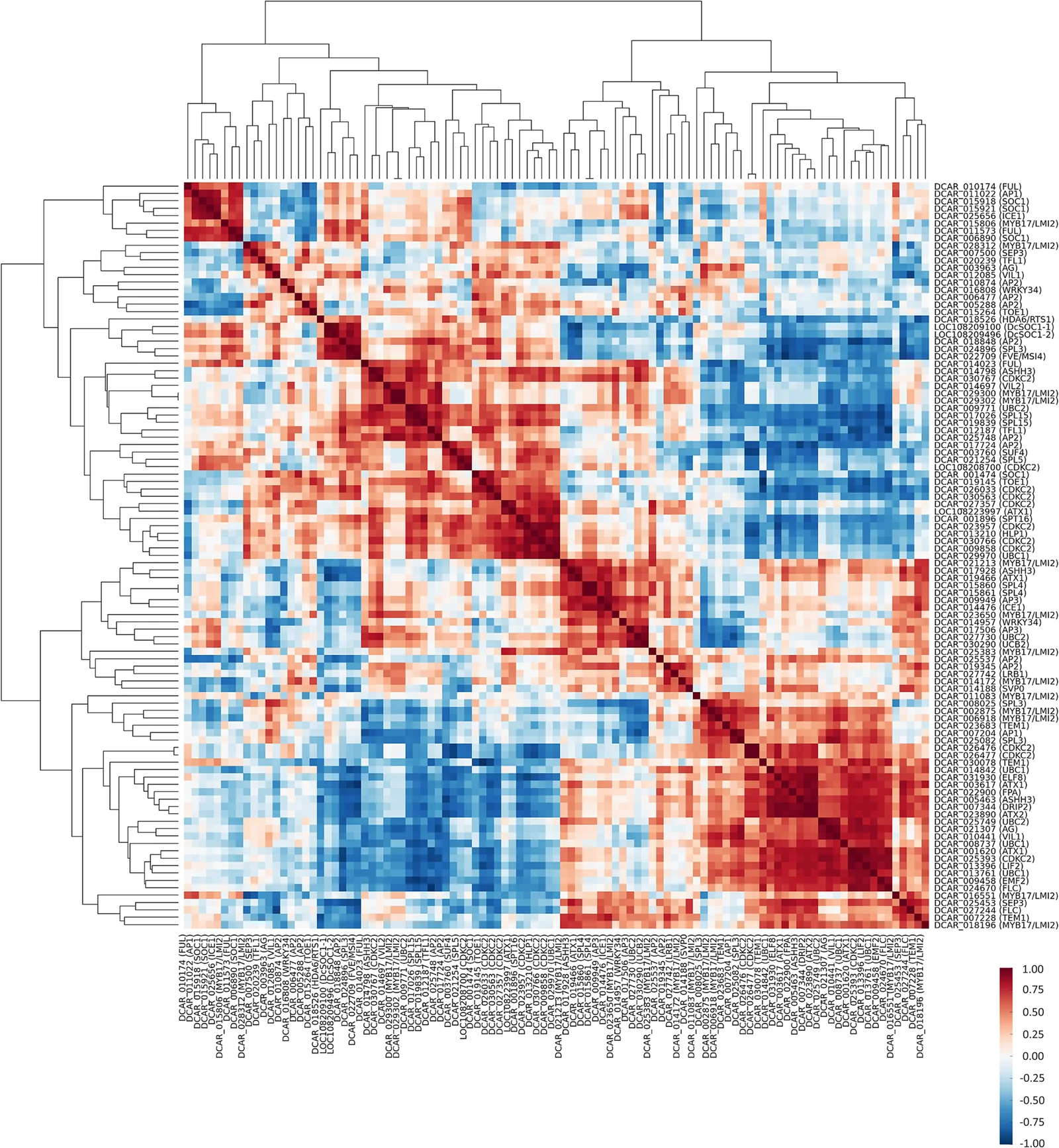
Pearson correlation coefficient heatmap of expression data (log CPM) for 102 carrot homologs of Arabidopsis genes involved in vernalization and flowering development pathways. Genes were hierarchically clustered using average linkage and Euclidean distance. Darker red and blue indicate stronger positive and negative correlation coefficient values, respectively

Lists of DEGs identified across different physiological/developmental stages within each carrot accession, and between carrot accessions for each physiological stage, are presented in Tables S14 and S15, respectively. Thus, intra-accession transcriptome comparisons among different physiological stages revealed 2,825 − 18,639 DEGs in B5, of which 1,598 − 10,045 genes were upregulated and 1,443-8,594 were downregulated (for each comparison, the least vernalized sample was always considered as reference); whereas for Criolla INTA, 2,361-8,089 DEGs were identified, of which 977-4,567 were upregulated and 1,507-3,522 were downregulated (Table S16). The comparisons with largest number of DEGs were those between any of the pre-flowering stages (i.e., Juv, Ctl, 40d, and 80d) vs. post-flowering (Flw), revealing 14,397 (in Ctl/Flw comparisons) to 18,639 DEGs (in 80d/Flw) in B5, suggesting a strong shift in global gene expression patterns associated with flowering. In general, comparisons among samples in Criolla INTA revealed less DEGs than in B5, which is likely due to the absence of Flw samples in the former cultivar. Among the DEGs identified from samples comparisons within B5 and within Criolla, 3–55 and 5–28 DEGs were in the *Vrn1* region, respectively; and, of those, 0–3 genes -depending on the comparison- had annotations related to vernalization and/or flowering pathways, and they correspond to the *Vrn1* candidates *DcSOC1-1*, *DcSOC1-2*, and *DcBAG6.* Transcriptome comparisons between B5 and Criolla INTA at each physiological stage revealed 8,238 − 12,480 DEGs, of which 17–50 DEGs were in the *Vrn1* region, and only one of them, namely *DcSOC1.1*, had annotations associated with vernalization and/or flowering pathways (Table S17). The expression profiles of the genes found in the *Vrn1* region that had annotations related to vernalization/flowering were described above and shown in Fig. 1E. We identified 217 carrot genes homologous to 68 Arabidopsis genes involved in the vernalization and flower development pathways (Table S7). Of these, 102 genes were differentially expressed in CTP vs. NVP comparisons within B5 and/or Criolla INTA (Fig. 3). Besides the *Vrn1*-candidates *DcSOC1-1* and *DcSOC1-2*, four SOC1 genes (DCAR_006890, DCAR_001474, DCAR_015918, DCAR_015921) had expression patterns consistent with their proposed role as flowering integrators -i.e., exhibiting upregulation in CTP- in B5 and two of them (DCAR_006890 and DCAR_001474) in Criolla INTA. Interestingly, one of these SOC1 genes, namely DCAR_006890, showed a remarkably high upregulation in CTP of both carrot lines, being its upregulation comparable to -and even greater- than for *DcSOC1-1* and *DcSOC1-2*, as indicated by the 11.3-107.5 and 39.4–55.6 fold greater expression level found for this gene in CTP compared to NVP samples of B5 and Criolla INTA, respectively. Other MADS-box TFs important for transitioning from vegetative to reproductive meristem are members of the AP1/FUL subfamily of MADS-box genes, particularly the paralogs FRUITFUL (FUL) and APETALA 1 (AP1) genes, which act as flowering activators, thereby promoting flowering time [62, 63]. We identified three FUL homologs (DCAR_011573, DCAR_010174, and DCAR_014023) upregulated in CTP of both carrot lines; one of them (DCAR_011573) exhibiting strong upregulation -in the range of 9.5-115.9 and 11.8–90.3 fold greater- relative to NVP of B5 and Criolla INTA, respectively. In general, these FUL genes had expression profiles that paralleled those of SOC1 DEGs. Additionally, one AP1 homolog (DCAR_011022) was upregulated in CTP of both carrot lines, showing concomitant expression patterns with those of FUL and SOC1 DEGs. Overall, the expression profiles of *DcSOC1-1*, *DcSOC1-2*, and different members of the flowering activators FUL, SOC1, and AP1 were positively correlated (Fig. 4), being the strongest associations those among *DcSOC1-1*, *DcSOC1-2*, DCAR_015918 (SOC1), DCAR_015921 (SOC1), DCAR_006890 (SOC1), DCAR_014023 (FUL), DCAR_010174 (FUL), DCAR_011573 (FUL), and DCAR_011022 (AP1), exhibiting correlation coefficient (r) values in the range of 0.50–0.97.

In carrot, two genes annotated as FLC, namely DCAR_024670 and DCAR_027244, were differentially expressed in CTP vs. NVP comparisons, exhibiting downregulation in the meristems of plants that underwent cold treatment. Thus, DCAR_024670 was significantly downregulated in all the cold-treated samples (40d, 80d, Flw) of B5 and in 80d-samples of Criolla INTA, relative to their NVP; whereas DCAR_027244 was significantly downregulated in 40d and 80d of B5, relative to Ctl. In Criolla INTA, the latter gene was also downregulated in both cold-treated samples, but without reaching statistical significance. Besides the major flowering repressor FLC, other genes can also repress flower initiation by different mechanisms. For example, ‘EMBRYONIC FLOWER 2’ (EMF2) encodes a transcription factor that participates in the polycomb group (PcG) protein complex-mediated silencing of flower homeotic genes, thereby repressing the floral program to promote vegetative growth in Arabidopsis [64]. Another type of flowering repressor genes is ASHH3, a histone methyltransferase that functions as a negative regulator of the vernalization process by altering histone methylation patterns in flowering-related genes [65]. We identified two ASHH3 (DCAR_017928 and DCAR_005463) and one EMF2 (DCAR_009458) genes that appear to be downregulated after cold treatment, relative to untreated controls, in both carrot lines, consistent with their predicted function as flowering repressors. The expression profiles of these genes were rather similar to those of the two FLC genes, an observation that was confirmed by the positive correlations found among them (Fig. 4; *r* = 0.45–0.86). As expected, based on their putative role as flowering repressors, the two FLC genes had general expression patterns inversely correlated with those of the flowering promoters *DcSOC1-1* (*r* = -0.36 to -0.40), *DcSOC1-2* (*r* = -0.26 to -0.42), DCAR_001474 (SOC1) (*r* = -0.65 to -0.70), and DCAR_006890 (SOC1) (*r* = -0.11 to -0.27); and so did the ASHH3 and EMF2 genes (*r* = -0.27 to -0.67) (Fig. 4). The expression profiles of other carrot homologs of Arabidopsis vernalization genes and their correlations can be found in Figs. 3 and 4.

### RT-qPCR analysis of genes associated with carrot vernalization and flowering

**Fig. 5:**
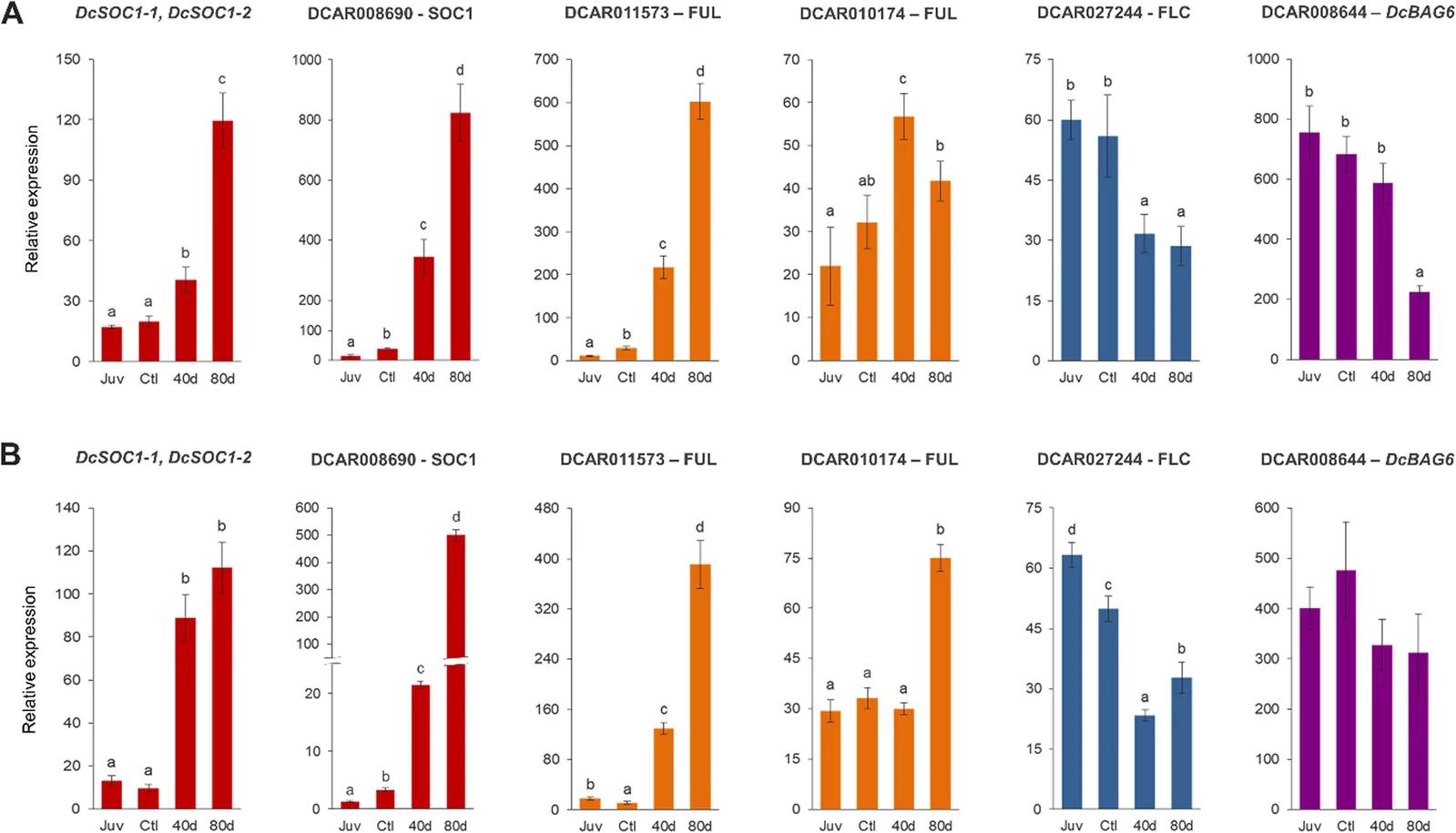
RT-qPCR expression profiles of *Vrn1*-candidate genes *DcSOC1-1*, *DcSOC1-2*, and *DcBAG6*; and three other genes potentially involved in carrot vernalization and flowering pathways (DCAR_011573, DCAR_010174, DCAR_027244) in the apical meristems of non-vernalized plants (Juv, Ctl) and cold-treated plants (40d, 80d) of the biennial carrot ‘B5’ (**A**) and the annual ‘Criolla INTA’ (**B**). DCAR_011573 and DCAR_010174 encode putative FRUITFUL (FUL) proteins [synonym of AGAMOUS-like 8 (AGL8)], and DCAR_027244 is annotated as FLOWERING LOCUS C (FLC). Graphs of flowering promoter and flowering repressor genes are depicted in warm and cold colors, respectively. Bars indicate mean values for the fold change in gene expression relative to the expression of the internal control gene (actin), as calculated from the ratio ∆Cq-target gene/∆Cq-actin [66] and their standard errors. Bars sharing the same letter are not significantly different at *p* ≤ 0.05. Graphs without bar letters indicate no statistical differences

Figure 5 presents gene expression analysis by RT-qPCR in three candidate genes identified in the *Vrn1* region –i.e., *DcSOC1-1*, *DcSOC1-2*, and *DcBAG6*- and four other genes potentially involved in vernalization and flowering. The latter were selected based on their annotations, which were directly related to these pathways, and the facts that they were differentially expressed in CTP vs. NVP comparisons in the RNA-Seq data, and their expression patterns across different carrot physiological stages agreed with their proposed annotated functions. These were DCAR_008690, encoding a SOC1 gene; DCAR_010174 and DCAR_011573, both encoding putative FRUITFUL (FUL) genes; and DCAR_027244, annotated as FLOWERING LOCUS C (FLC). Given the high sequence similarity between *DcSOC1-1* and *DcSOC1-2* at the mRNA level (99% identity, with only 10 SNPs differentiating mRNA molecules from both genes), the primers used for RT-qPCR analysis do not differentiate expression levels from these two genes (i.e., the combined expression levels of *DcSOC1-1* and *DcSOC1-2* were estimated). In both carrot lines, the expression patterns of *DcSOC1-1* and *DcSOC1-2* coincided with their expression profiles based on transcriptome analysis (Fig. 1E), revealing significant upregulation in CTP exposed to 40 and 80 days of cold, as compared to NVP (Juv, Ctl). The relative increase in expression levels in CTP vs. NVP were 6.1–11.1 fold in B5, and 2.1-7.0 fold in Criolla INTA, indicating a strong activation of these genes after prolonged cold exposure. For the other SOC1 gene (DCAR_008690), a very similar expression patterns was observed, but exhibiting even stronger upregulation in CTP relative to NVP, with relative increases in expression levels of 9.0-51.2 and 6.5-394.4 fold in CTP of B5 and Criolla INTA, respectively.

The two putative FUL genes exhibited expression patterns that were compatible with their role –in Arabidopsis- as flowering promoters that regulate the transition from vegetative to reproductive state in the apical meristem [63]. These two transcription factors were significantly upregulated in CTP exposed to 40 and/or 80 days of cold, as compared to NVP. The extent of upregulation in CTP was noticeably greater for DCAR_011573, with increases in expression levels in the range of 7.2–51.0 and 6.9–35.2 in B5 and Criolla INTA, respectively; whereas DCAR10174 showed weaker upregulation in CTP, with increases of 1.3–2.6 in both carrot lines. Overall, the RT-qPCR expression patterns of these putative FUL genes were comparable to those found by RNA-Seq analysis (Fig. 3).

Expression analysis of DCAR27244, encoding a putative FLC gene, exhibited an expression profile compatible with its reported role in Arabidopsis. FLC is a MADS-box transcription factor that acts as a floral repressor, being highly expressed in the vegetative meristems (before cold exposure), whereas during and after cold exposure, the gene is epigenetically and quantitatively repressed, thereby allowing the upregulation of flowering-promoting genes [8]. In carrot meristems, this gene was highly expressed in NVP vegetative actively-growing juvenile and control plants, but it was significantly downregulated in CTP after 40 and 80 days of cold, in both the annual and biennial carrot lines. Lastly, *DcBAG6*, a putative flowering repressor gene, revealed high expression levels across most samples of both carrot accessions, being differences only significant in B5, for Juv, Ctl, and 40d samples relative to 80d, whereas no statistical differences were observed among physiological stages of Criolla INTA.

### Characterization and expression analysis of noncoding transcripts

**Fig. 6:**
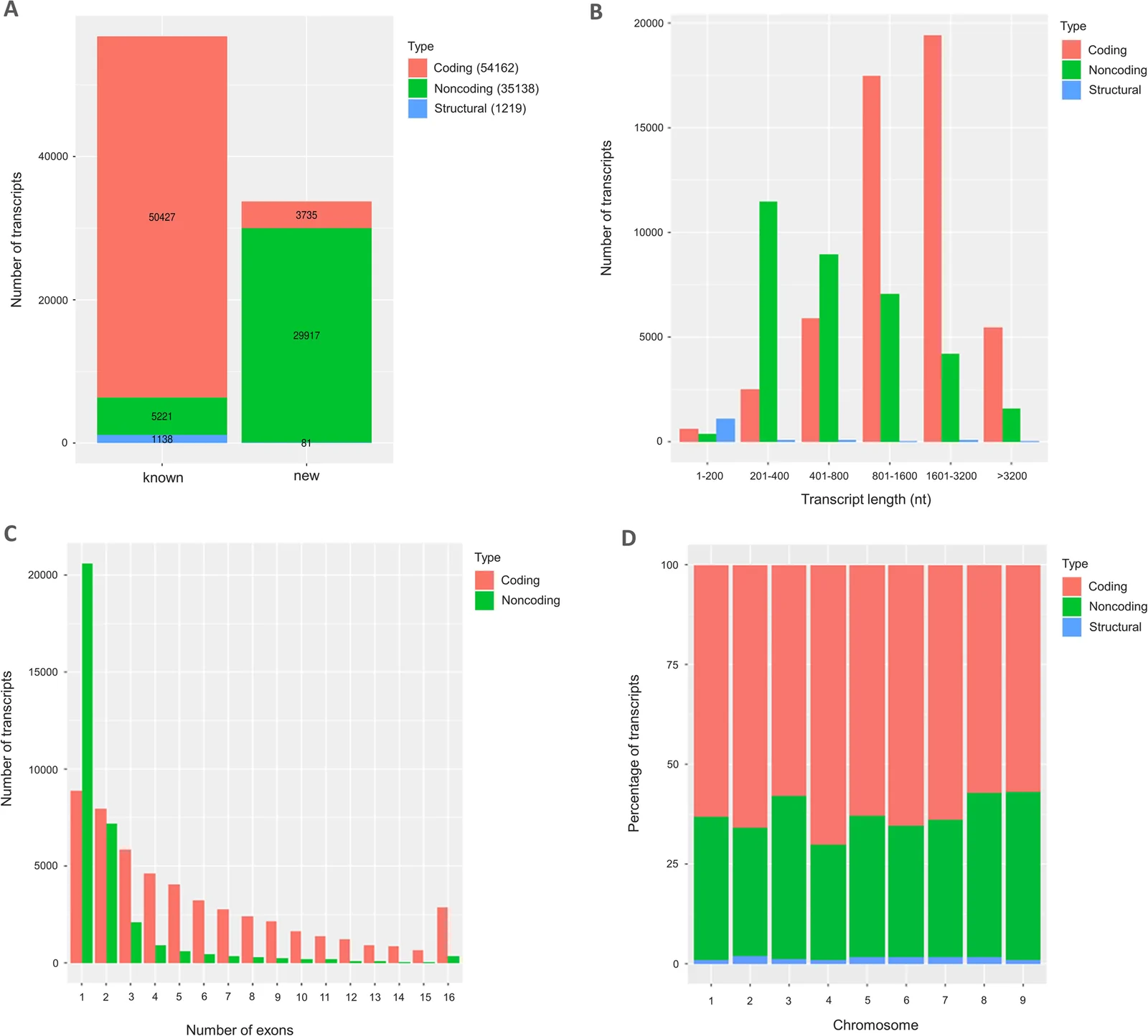
Characterization of carrot transcripts from apical meristems. **A** Distribution of coding, noncoding, and structural between the known and newly annotated transcripts. **B** Transcript length distribution for the coding, noncoding, and structural RNAs. **C** Number of exons per transcript for the coding and noncoding RNAs. **D** Percentual distribution of the coding, noncoding, and structural RNAs across the chromosomes

Characterization of the transcripts from carrot apical meristem, regardless of accession and physiological status, revealed a total of 54,162 coding transcripts, 35,138 noncoding transcripts, and 1,219 structural transcripts (Fig. 6). Of these, 33,734 were new transcripts, and comprised 3,735 new protein-coding and 29,999 non-coding transcripts (1,606 lncNATs, 28,312 lincRNAs and 81 structural transcripts). The list of new transcripts and their annotation in presented in Table S18. These add to the set of 56,786 already known transcripts, including 50,427 coding transcripts and 6,359 non-coding transcripts (Fig. 6A). Most non-coding mRNAs presented less than 800 nt long, being 201–400 nt the most frequent length class (Fig. 6B), and the same was found for the subset of new noncoding transcripts (Fig. S9A). Coding transcripts between 1,601 and 3,200 nt long were the most frequent, while most structural transcripts presented 200 or less nt. Noncoding transcripts predominantly presented a single exon and, unexpectedly [67], only one exon was also the most frequent class for coding transcripts (Fig. 6C). Additionally, we found no particular bias for the distribution of the noncoding transcripts along the nine carrot chromosomes (Fig. 6D). In the subset of newly-identified transcripts, these features were generally comparable to the population of known transcripts (Fig. S9B-C).

A total of 5,410 ncRNAs were differentially expressed across different physiological/developmental stages within B5 and within Criolla INTA, of which 4,853 (89.7%) were differentially expressed specifically in pair-wise comparisons of NVP vs. CTP samples. Thus, depending on the comparison, 319-1,497 noncoding transcripts were upregulated and 227-1,598 were downregulated in CTP relative to NVP (Tables S19 and S21). Comparisons between the two carrot lines at each of the physiological/developmental stages revealed, overall, 4,722 differentially expressed ncRNAs; of which 3,059 transcripts (64.5%) were differentially expressed between CTP samples of the two lines. Of these, 1,275 noncoding RNAs were upregulated and 1,784 were downregulated in B5 relative to Criolla INTA (Tables S20 and S21). Despite these general differences in expression patterns of noncoding RNAs between CTP and NVP, and between the biennial and annual carrot lines, we did not find differentially expressed ncRNAs directly related to the *Vrn1* candidates *DcSOC1-1*, *DcSOC1-2*, and *DcBAG6*. Furthermore, no differentially expressed ncRNAs related to any of the carrot homologs to Arabidopsis vernalization/flowering genes (Table S7) were identified.

## Discussion

The genetics of vernalization requirement has been studied in several dicots, including *Hyoscyamus niger* [68], *Beta vulgaris* [69], some *Brassica* species, e.g., *B. napus* [70, 71] and *B. rapa* [72–74], and Arabidopsis [6, 75–78]; as well as in some monocots such as wheat [79] and barley [80]. In the model Arabidopsis and some major crops (e.g., wheat and barley), the knowledge on vernalization genetics has reached extensive detail, unveiling functionally-characterized key genes and the molecular mechanisms by which these genes interact and respond to environmental and endogenous stimuli [1]. Conversely, in carrot, little is known about the genetic regulation of VR and the genes conditioning this trait have not been identified to date. Previous inheritance and linkage analyses revealed a single dominant locus conditioning VR, termed *Vrn1*, positionally located at the end of Chr. 2 in and AFLP-based map [26, 27]. In the present work, we used a large F_2_ population (*N* = 1000) derived from the same genetic background used earlier [26, 27] to phenotype and genetically map VR. This led to the identification of a simply-inherited locus controlling VR, dominant for the annual flowering habit over the biennial habit, and its mapping at the distal end of Chr. 2, in a high-density GBS-derived SNP-based linkage map (Fig. 1A). Because we used the same genetic background, and both the phenotypic segregation ratios (3:1 annual: biennial) and map position of VR coincided with those reported earlier [27], we adopted the same term, *Vrn1*, for this locus. The immediate flanking markers of *Vrn1* spanned a 1.9 cM region, representing ~ 187,243 nt of the carrot genome sequence (from SNP markers 43,471,032 to 43,658,275). Additional GWAS analysis revealed a broader region with highly significant marker-trait associations that overlapped *Vrn1*, spanning ~ 694 kb of the genome sequence (from 43,130,258 to 43,824,418) (Fig. 1B); which was used to search for *Vrn1*-candidate genes. The current linkage map and GWAS analyses represent a substantial improvement in terms map saturation and resolution, and marker informativeness, for the *Vrn1* region, as compared to our previous map [27].

Two SOC1 genes, termed *DcSOC1-1* and *DcSOC1-2*, were identified in the *Vrn1* region (Fig. 1C) and they consistently exhibited significant and strong upregulation in CTP relative to NVP, in both B5 and Criolla INTA (Fig. 1E), in agreement with their annotated function as flowering promoter genes, which in Arabidopsis are typically upregulated upon vernalization [1]. In addition, the two SOC1 genes were phylogenetically clustered with functionally-characterized SOC1 genes from other species (Fig. 1D). Lastly, the expression patterns of these genes across CTP and NVP samples of both carrot lines were confirmed by means of RT-qPCR analysis (Fig. 5). Altogether, these results fulfilled the four criteria for declaring *Vrn1* candidates, as described in materials and methods section. Lastly, *DcSOC1-1* and *DcSOC1-2* showed positively-correlated expression patterns with other genes known to promote vernalization and flowering in Arabidopsis, including other SOC1 genes (DCAR_006890, DCAR_001474, DCAR_015918, DCAR_015921), and homologs of FUL (DCAR_011573, DCAR_010174, and DCAR_014023) and AP1 (DCAR_011022); and they were negatively correlated with the expression patterns of flowering-repressor genes, such as FLC (DCAR_024670 and DCAR_027244), ASHH3 (DCAR_017928 and DCAR_005463) and EMF2 (DCAR_009458) homologs (Figs. 3, 4 and 5). These data provide further evidence for the candidacy of *DcSOC1-1* and *DcSOC1-2* as the genetic determinants of *Vrn1*.

Comparison between the two carrot lines for the expression profiles of *DcSOC1-1* and *DcSOC1-2* revealed that *DcSOC1-1* was significantly upregulated (*p* < 0.01) in plants of the annual carrot Criolla INTA exposed to 80 days of cold as compared to plants of the biennial B5 that received the same cold treatment. The former had a nearly 4-fold greater expression level than in 80d-samples of B5 (Fig. 1E). After 40 days of cold, Criolla INTA also had greater expression level for *DcSOC1-1* (2.7-fold greater) than B5, but this difference did not reach statistical significance. No differences were observed between the two carrot lines for *DcSOC1-2*. These data suggest that *DcSOC1-1*, but not *DcSOC1-2*, is responsible for differences in VR between annual and biennial carrots, as annual carrots evidenced a greater transcription rate of *DcSOC1-1* under cold exposure, presumably allowing them to reach a vernalized state earlier than for biennials. We may speculate on two different ways by which *DcSOC1-1* (but not *DcSOC1-2*) confers VR differences between annuals and biennials; (i) the two putative SOC1 genes may have functionally different roles, being *DcSOC1-1*, but not *DcSOC1-2*, the actual floral integrator that promotes flowering; (ii) both genes have identical SOC1 roles but, under cold exposure, *DcSOC1-1* is transcribed more actively in annuals than in biennials, increasing the general pool of SOC1 transcripts faster in annuals and allowing them to surpass a critical threshold -needed to achieve a vernalized state- earlier. However, considering the high sequence similarity at the predicted protein level between the two SOC1 genes (98% identity), it is unlikely that they will have different functions. In contrast, following the ‘SOC1 transcripts dosage’ hypothesis, when adding up the normalized read counts of both genes (i.e., *DcSOC1-1* + *DcSOC1-2*), it was found that, after 40 days of cold, Criolla INTA had 4.2 times greater transcript levels than B5, and 1.6 times greater after 80 days of cold, suggesting that, when exposed to cold, annual carrots reach high SOC1 transcript levels faster than biennials, possibly explaining VR differences between the two. Results from the RT-qPCR analysis of the two SOC1 genes, indicating greater transcript levels in Criolla than in B5 after 40 days of cold, generally agreed with these data (Fig. 5). Additional studies aiming at functionally-characterizing *DcSOC1-1* and *DcSOC1-2* are needed to test these hypotheses and elucidate the molecular mechanism by which they condition VR in annual and biennial carrots. Such studies should also include the comparative analysis of the early-flowering (from Criolla INTA) vs. late-flowering (B5) alleles of *DcSOC1-1* and *DcSOC1-2* to identify the causal mutations responsible for VR differences between annual and biennial carrots. The differences is number and length of MITE insertions observed in the upstream region of both genes (i.e., three small insertions in *DcSOC1-1* vs. one larger insertion in *DcSOC1-2*), relatively close to the transcription start site, are structural variants that, due to their position, may emerge as relevant for explaining expression patterns differences between the two genes.

The MADS-box gene *DcAGL6*, identified in the *Vrn1* region, was discarded as a potential candidate because it was not differentially expressed for all the comparisons considered, including CTP vs. NVP. Additional comparative analysis of this gene between B5 and Criolla revealed 100% sequence identity at the mRNA and predicted protein levels, indicating high-sequence conservation for *DcAGL6* between the two accessions. These results further support the elimination of this gene as a *Vrn1*-candidate in these genetic backgrounds.

In the *Vrn1* region we identified gene DCAR_008644, annotated as ‘BAG family molecular chaperone regulator 6 (Bcl-2-associated athanogene 6)’ and termed *DcBAG6*, which predicted protein sequence was phylogenetically related to BAG6 proteins from other species, including *atbag6*, from Arabidopsis (Fig. S4). This class of BAG proteins contains a calmodulin-binding domain/isoleucine glutamine (IQ motif) by which they can interact with calmodulin (CaM) proteins to exert numerous biological processes, from plant growth and development to biotic and abiotic stress responses [81]. In Arabidopsis, knock-out mutants of *atbag6* resulted in early flowering and branched inflorescences, suggesting that this gene may act as a flowering repressor [61]. However, in grape, overexpression of *HSG1*, a BAG-encoding gene with similar protein sequence to those of *atbag5* and *atbag6*, showed faster floral transition by activating the expression of CONSTANS (CO), thereby acting as a flowering-promoting gene [82]. Besides these two reports, we could not find other studies directly associating BAG6 genes with vernalization or flowering time. In this study, *DcBAG6* was highly expressed throughout all physiological/developmental stages in B5 and Criolla, but showed significant downregulation in CTP relative to NVP in both carrot lines (Fig. 1E), suggesting a role as a negative flowering regulator, similar to that of *atbag6*. These expression patterns were not fully validated by RT-qPCR analysis (Fig. 5). Thus, despite its map colocalization with *Vrn1* and expression profiles showing weak -yet significant- downregulation in CTP, the role of *DcBAG6* in carrot VR remains inconclusive and warrants further research.

The present study identified a simply-inherited locus, *Vrn1*, with two tightly-linked duplicated SOC1 genes that are strong candidates for the genetic control VR. It must be noted that this work and the previous VR inheritance and mapping studies (Alessandro & Galmarini, 2007; Alessandro et al., 2013) used as plant materials the same genetic background, namely different segregating families (F_2_, BC_1_) derived from crosses between the annual Criolla INTA and the biennial B5. However, in another study that analyzed VR segregation ratios in several F_2_ families from different crosses of early-flowering × late-flowering accessions, we observed segregation ratios that were compatible with a digenic control of VR, with both loci being dominant for early-flowering over late-flowering [45]. In that study, an F_2_ population with the same genetic background as the one used in the present study (i.e., B5 × Criolla INTA) was included and showed a 3:1 (early-flowering : late-flowering) segregation ratio, suggesting a monogenic control of VR, consistently with the findings of the present work and those of Alessandro and Galmarini [26] and Alessandro et al. [27]. The 3:1 segregation ratio consistently found in F_2_s derived from ‘B5 × Criolla INTA’, along with the linkage mapping and GWAS results of this study, clearly indicating a single locus (*Vrn1*) conditioning VR, are compatible with a two-gene model, if the other locus is fixed (e.g., recessive or homozygous dominant). Also, phenotypic analysis of flowering rates in various early- and late-flowering accessions subjected to different lengths of cold exposure revealed differences between early and late flowering accessions, and also among accessions within these two groups, suggesting a gradient of VR in the carrot germplasm. Altogether, these data suggest that additional loci and genes -besides *Vrn1* and *DcSOC1-1* and *DcSOC1-2*- conditioning VR may be discovered in future studies using plant materials with other genetic backgrounds. To this end, linkage mapping approaches using F_2_ populations showing a clear digenic segregation ratio for VR, as some of those reported by Wohlfeiler et al. [45]; or GWAS analysis in a large and diverse global carrot germplasm collection (*N* = 695) characterized for flowering habit, as the one reported recently [17], would be ideal for identifying new loci and genes conditioning VR in carrot.

The molecular mechanisms of vernalization have been studied and described to extensive detail in Arabidopsis [1, 8, 83]. The results from this study, including the identification of *DcSOC1-1* and *DcSOC1-2*, along with other genes homologous to Arabidopsis key genes involved in vernalization (e.g., homologs of SOC1, FUL, AP1, FLC, ASHH3, EMF2, among others), all of them showing correlated expression profiles -across different CTP and NVP of both carrot lines- compatible with their presumed function as either flowering activators or flowering inhibitors (Figs. 1, 3, 4 and 5), suggest that the process of vernalization in carrot is governed by a similar system and set of genes as in Arabidopsis. However, our results indicate major differences between the two species for vernalization requirement. In Arabidopsis, genetic studies on variations in VR using natural accessions have demonstrated that this requirement is mainly due to two dominant flowering-repressor genes, FRIGIDA (FRI) and FLC [6, 84–89]. Because FLC proteins directly bind to downstream floral integrators, including FT, FD, and SOC1, to inhibit their transcription [90], high levels of FLC are responsible for VR in Arabidopsis [6, 87]. FRI mainly acts to upregulate FLC transcription and thus contributes to VR in Arabidopsis accessions [6]. Because of their flowering-inhibiting functions, naturally-occurring mutations in FRI and FLC are responsible for early flowering in many Arabidopsis accessions [77, 87, 89]. Thus, while in Arabidopsis VR is mainly conditioned by FLC and FRI, in carrot it is controlled by two SOC1 genes (*DcSOC1-1* and *DcSOC1-2*). This aligns with the fact that, in Arabidopsis, VR is dominant for late flowering, as a result of the flowering-repressing role of the genes conditioning this requirement, FRI and FLC, whereas in carrot VR is dominant for early flowering (this study; [26]), consistent with the flowering-promoting role of the two SOC1 genes controlling *Vrn1*, identified in the present work.

A very recent study evaluated flowering time and the expression of these two SOC1 genes in ‘Songzi’ (SZ), a wild species of carrot that does not require vernalization (i.e., it flowers without cold exposure), and the biennial carrot ‘Qitouhuang’ (QTH), reporting that *DcSOC1-2* (termed *DcSOC1d* in their study) from SZ promotes early flowering, and suggested *DcSOC1-2* as a candidate for *Vrn1* [29]. They observed that QTH plants transformed with *DcSOC1-2* from SZ (transformation constructs contained both the coding and promoter sequences) showed early flowering without the need of vernalization, whereas those transformed with *DcSOC1-1* (called *DcSOC1b* in their study) had the same flowering phenotype as the untransformed QTH plants (i.e., they did not flower without cold). Because *DcSOC1-1*-transgenic QTH plants had very low expression levels of both genes (*DcSOC1-1* and *DcSOC1-2*) but *DcSOC1-2-*transgenic lines showed upregulation of both *DcSOC1-1* and *DcSOC1-2*, they proposed that *DcSOC1-2* positively regulates the expression of *DcSOC1-1.* In addition, the authors hypothesized that a transposon insertion found in the promoter region of *DcSOC1-2* in QTH could be the cause of the vernalization-induced late-flowering phenotype in this biennial carrot. Although their findings, using different plant materials and methodological approaches, point at the same SOC1 genes as candidates for carrot VR, in agreement with results from the present work, some considerations and differences between the two studies must be noted. Firstly, the study of Zhang et al. [29] compared a wild carrot (*D. carota* spp. *carota*) with no VR and a typical biennial carrot, and mainly focused on the analysis of *DcSOC1-1* and *DcSOC1-2* from the wild early-flowering SZ, evaluated under non-vernalizing condition (i.e., without cold exposure) in the genetic background of SZ and -by transgenesis- QTH. Thus, the functional and structural analyses of these genes from SZ are not directly extrapolable to the role of *DcSOC1-1* and *DcSOC1-2* in a cultivated annual carrot, which typically requires 5–30 days of vernalizing temperatures for flowering [16, 18, 21]. In addition, cultivated carrots -annuals and biennials- have a prolonged juvenile stage during which they are not sensitive to the cold stimulus (vernalization), whereas in wild carrots there is almost no juvenile stage, exhibiting premature flowering in very young plantlets [14, 16, 22]. In contrast, the present study compared two cultivated carrot (*D. carota* ssp. *sativus*) lines, an annual and a biennial, both requiring vernalization to flower, but varying in VR. Thus, although *DcSOC1-1* and *DcSOC1-2* seem to account for VR differences between the early- and late-flowering phenotypes in both studies, the function and structure of both SOC1 genes in SZ [29] may vary from those in Criolla INTA (the present study). Secondly, in the wild carrot SZ, *DcSOC1-2* was expressed more intensively than *DcSOC1-1*, and the former was proposed to upregulate the expression of the latter, suggesting *DcSOC1-2* as the main candidate gene conditioning early flowering [29]. However, no evidence of physical interaction between *DcSOC1-1* and *DcSOC1-2* was provided and, as stated by the authors, there are no reports in the literature of a SOC1 gene regulating the expression of another SOC1. Conversely, in the present study, *DcSOC1-1* was the only SOC1 gene that was differentially expressed between the two carrot lines after cold exposure, being this gene significantly upregulated in the annual Criolla INTA relative to the biennial B5 (Fig. 1E), suggesting that *DcSOC1-1* (but not *DcSOC1-2*) promotes early flowering. Lastly, the present study provides strong genetic mapping data, by linkage and GWAS analyses, along with comparative transcriptomics, in the same genetic background where *Vrn1* was first described, allowing the positional and functional association of the candidate genes with *Vrn1*. The discrepancies between both studies are likely due to differences in the plant materials used, suggesting variation in the regulation of vernalization associated with the *Vrn1* locus among carrot accessions. Overall, results from the present study complement those of Zhang et al. [29], with both studies pointing at *DcSOC1-1* and *DcSOC1-2* as major determinants of VR in carrot. Additional research is required to functionally characterize these genes, ideally in different genetic backgrounds of annual and biennial carrots, to further elucidate the molecular mechanisms underlying vernalization requirement in this crop.

Overall, our data provide a framework for advancing research on the genetic and molecular mechanisms regulating carrot VR. For instance, the individual and additive effects of *DcSOC1-1* and *DcSOC1-2* on the regulation of VR can now be assessed by means of silencing (e.g., by means of CRISPR-Cas technology) or upregulating (via transgenics) the expression of these genes in annual and biennial carrots. Particularly, the generated sequence and gene expression data for Criolla INTA and B5 will facilitate the precise design of guide RNAs targeting these SOC1 genes, individually or combined, for the production of knock-out mutants in these genetic backgrounds well-characterized for their flowering habits and responses to cold exposure. Alternatively, or in parallel, the role of these genes can be assessed by inducing their overexpression -by transgenic research- and observing their effect on vernalization-induced flowering time, as compared to untransformed controls. The regulatory role of the promoter regions of *DcSOC1-1* and *DcSOC1-2* in annual and biennial carrots should also be addressed in future studies, as this may help explain different expression patterns of these genes and their effects on VR. For instance, comparative analysis of TF binding sites in the promoters of both SOC1 genes in both accessions, followed by validation *via* protein-DNA binding experiments (e.g., yeast one-hybrid) and in vivo reporter gene assays may help dissect the role of these SOC1 genes in regulating VR. Besides dissecting the precise role of *DcSOC1-1* and *DcSOC1-2* in the genetic control of *Vrn1*, future studies of this kind, involving GWAS or linkage mapping in other genetic backgrounds, combined with transcriptome analysis, may reveal new loci controlling VR.

## Conclusions

By integrating inheritance, linkage mapping, marker-trait association, phylogenetic, and transcriptome analyses of coding and non-coding RNAs, two major candidate genes conditioning VR, *DcSOC1-1* and *DcSOC1-2*, were identified, and their expression profiles were characterized across various developmental and physiological stages related to vernalization. Structural analysis revealed that these are duplicate genes with high sequence identity at the mRNA and protein levels, showing also conserve sequences between the accessions, and they likely have redundant roles as flowering integrators in the vernalization pathway. However, *DcSOC1-1* showed earlier and stronger upregulation in the annual carrot compared to the biennial carrot, suggesting that it may be the one responsible for the early-flowering phenotype. Mechanistically, we hypothesize that the earlier upregulation of *DcSOC1-1* in annual carrots may raise the level of total SOC1 proteins to surpass a critical threshold required for vernalization before than in biennial carrots. Altogether, the data from the present study sets the stage for advancing research into the molecular mechanisms regulating carrot VR. From a breeding perspective, the discovery and potential manipulation of genes controlling vernalization-induced flowering time is expected to have a positive impact in improving root and seed production in carrot.

## Supplementary Information

Supplementary Material 1.

Supplementary Material 2.

## Data Availability

Phenotypic (VR) and genotypic (GBS) data for linkage mapping and GWAS are included as Supporting Information: Additional File 1, Tables S8 and S10. Raw sequencing reads have been archived on NCBI under project PRJNA1391034 (http://www.ncbi.nlm.nih.gov/bioproject/1391034).
