# Supplementary material for: A pair of duplicated SOC1 genes, DcSOC1-1 and DcSOC1-2, control vernalization requirement in carrot
Source: BMC Plant Biol. 2026 Aug 8;26:1910. doi: 10.1186/s12870-026-09529-z (PMC13746629; doi:10.1186/s12870-026-09529-z)
Supplement: Supplementary file 1 [file 12870_2026_9529_MOESM2_ESM.pdf]

SUPPLEMENTARY FIGURES

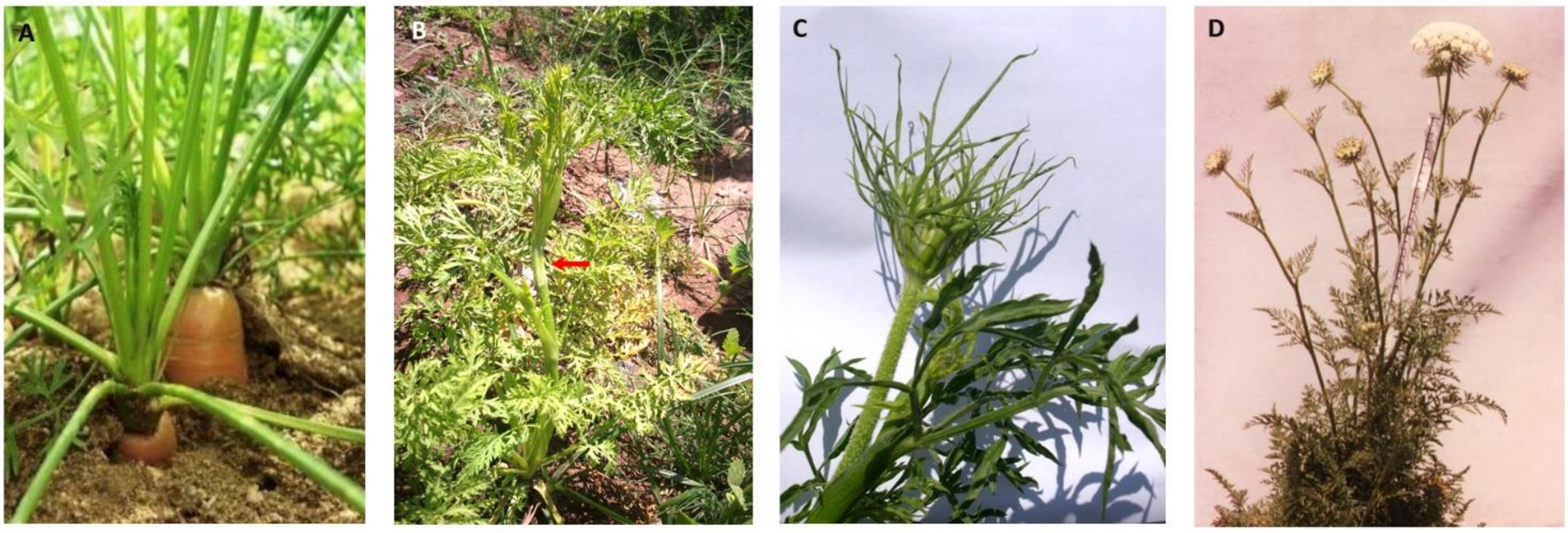

Fig. S1 Phenological stages of carrot flowering plants. (A) Plant in vegetative (non-flowering) stage; (B) first floral stem internode elongated (arrow indicates the floral stem); (C) floral primordia; (D) plant with primary umbel fully flowered.

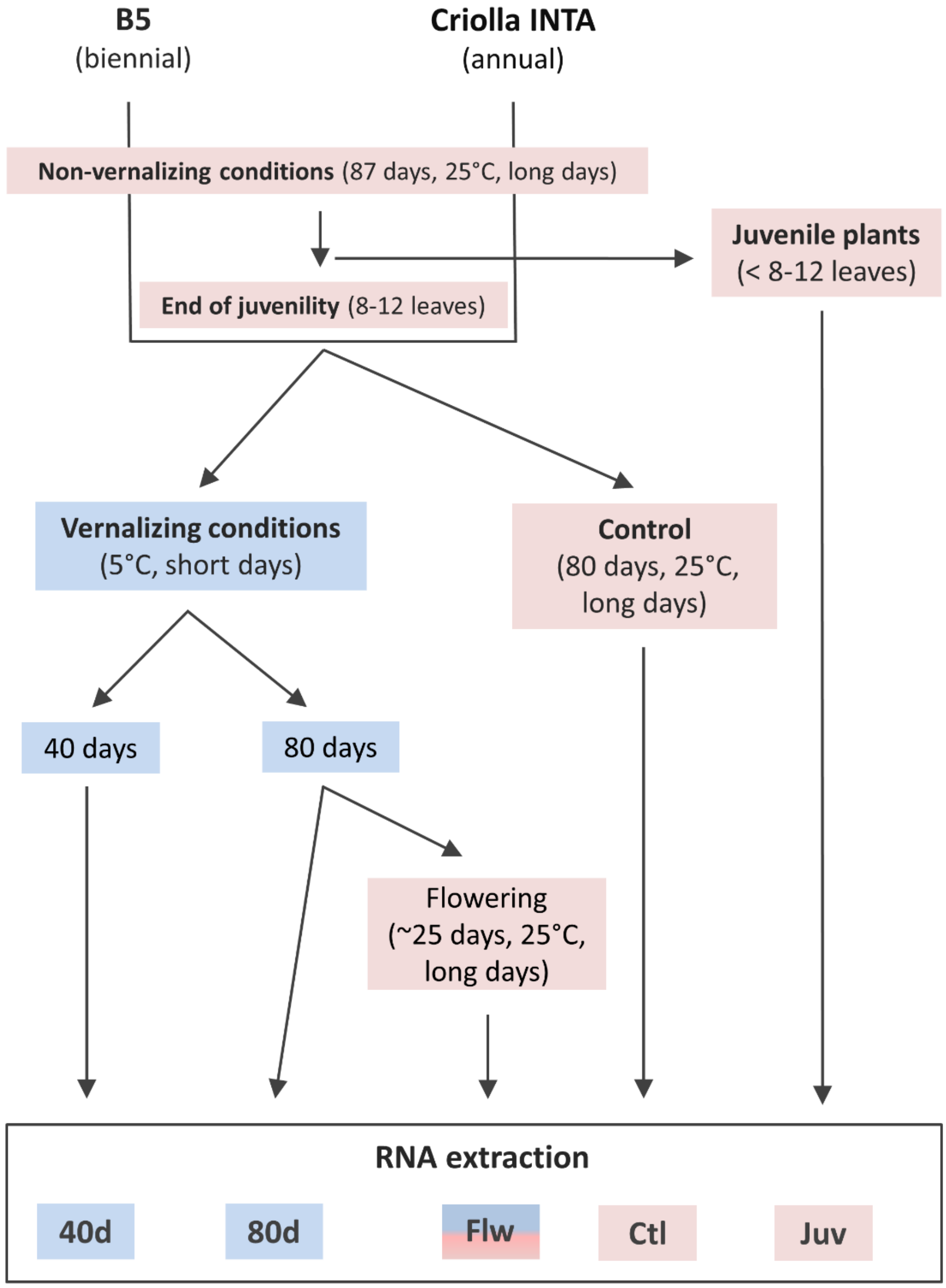

Fig. S2 Experimental design and treatments used for comparative transcriptome analysis of plants of the annual (Criolla INTA) and biennial (B5) carrot lines grown under vernalizing (blue boxes) and non-vernalizing conditions (pink boxes). As result of this experiment, the apical meristems from 15-20 plants of each carrot line were dissected and combined, in pools of five meristems each, at each of five time-points, representing five physiological stages [i.e., juvenility (Juv), control (Ctl), after 40 days of vernalizing conditions (40d), after 80 days of vernalizing conditions (80d), and after flowering (flw)]. The samples (3-4 biological replicates per accession and treatment) were used for RNA extraction, sequencing, and transcriptome analysis.

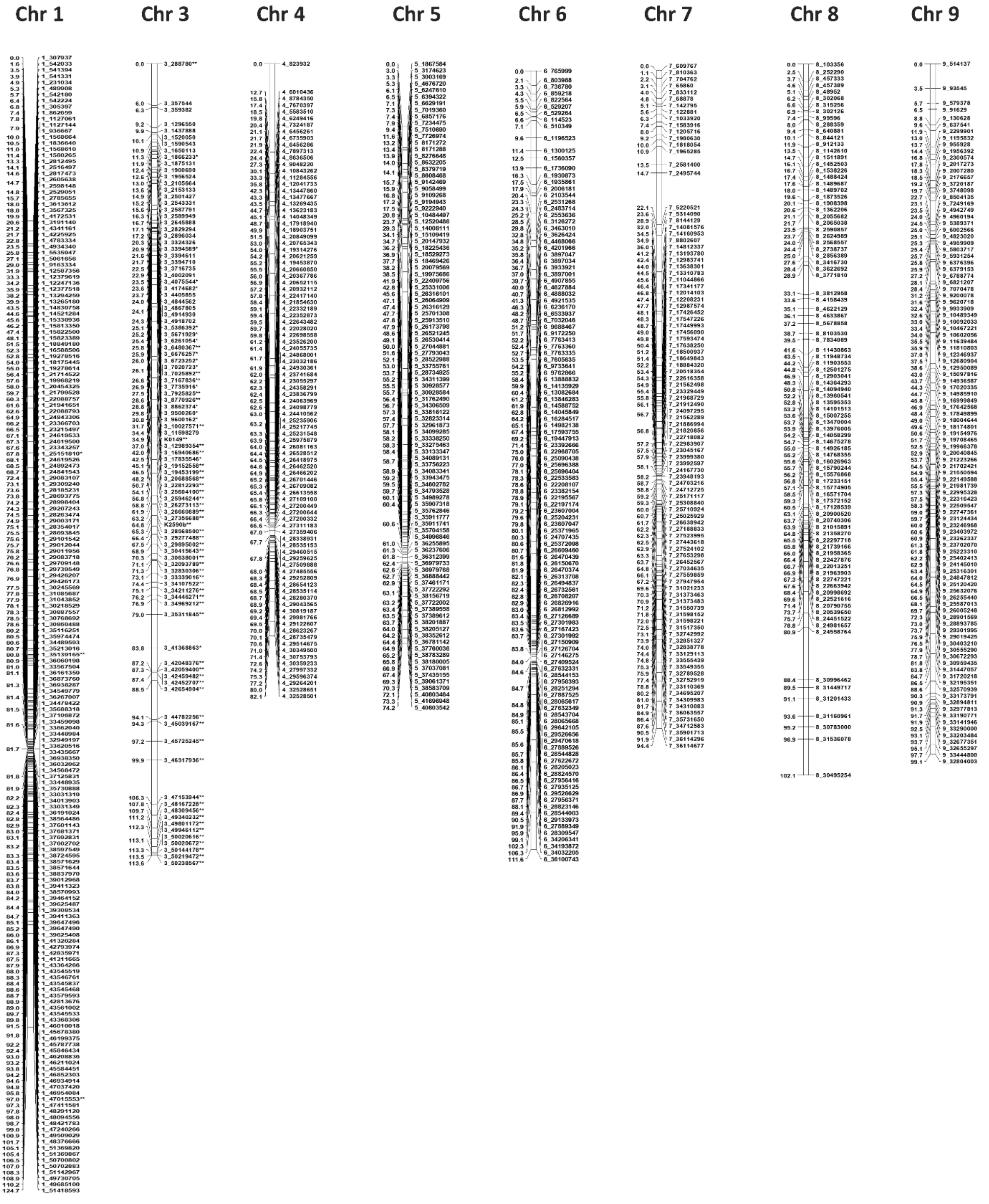

Fig. S3 Genetic linkage map constructed from the F_2 population derived from a cross between the annual Criolla INTA and the biennial B5 carrot lines. Linkage groups (LGs) were constructed using a LOD value ≥10. LGs were named according to their corresponding chromosomes (Chr). Assignment of the LGs to the chromosomes was done through physical localization of the GBS markers in the annotated carrot genome sequence. The map of Chr 2, containing *Vrn1*, was presented in Figure 1 of the manuscript. Additional characteristics of the genetic map are presented in Supplementary Table S9.

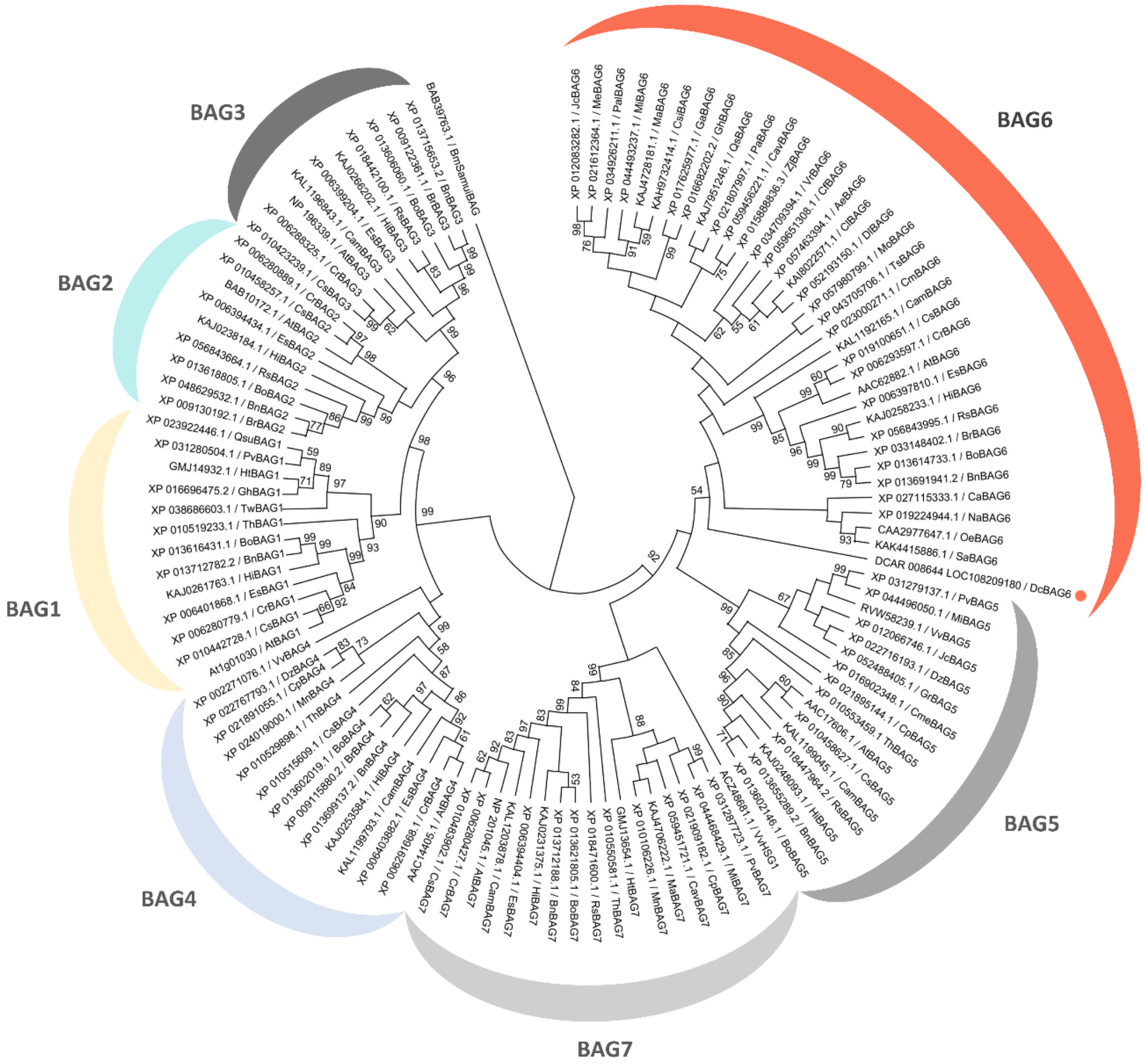

Fig. S4 Phylogenetic relationships among the predicted protein sequence of the carrot *DcBAG6* gene and 116 protein sequences of the Bcl-2-associated anthanogene (BAG) gene family with known functions. The phylogenetic cluster comprising BAG6 proteins and *DcBAG6* are indicated with an orange arc and circle, respectively. Bootstrap values ≥ 50 are indicated. More information on the sequences and species used is presented in Supplementary Table S3.

Comparison at the mRNA level of *DcSOC1-1* and *DcSOC1-2* in the reference carrot genome of DH1

Query: *DcSOC1-1* (LOC108209100)
Subject: *DcSOC1-2* (LOC108209496)

NW Score	Identities	Gaps	Strand	Frame
1774	945/994(95%)	41/994(4%)	Plus/Plus	

```
Query  1    -----------------------------GGGCTTGTTCTGTGCTCTCTTGGAAATAATT  31
                                         |||||||||||||||||||||||||||||||
Sbjct  1    CAAAACTTACCTTTGTTTCTACCATTCTTGGGCTTGTTCTGTGCTCTCTTGGAAATAATT  60

Query  32   TGGGATCTCTCTTCCCCTTTATAAAGTTGTTTCTTTGCCTTTTTTAGGCCTATCTTTCCT  91
            ||||||||||||||||||||||||||||||||||||||||||||||||||||||||||||
Sbjct  61   TGGGATCTCTCTTCCCCTTTATAAAGTTGTTTCTTTGCCTTTTTTAGGCCTATCTTTCCT  120

Query  92   CCACCTAAATCATCTCTTCTCTTGTTTCTTTTTCCATTTCCTCTTCTGTTGTGGAGATCG  151
            ||||||||||||||||||||||||||||||||||||||||||||||||||||||||||||
Sbjct  121  CCACCTAAATCATCTCTTCTCTTGTTTCTTTTTCCATTTCCTCTTCTGTTGTGGAGATCG  180

Query  152  AAAATGGTGAGGGGGAAGACTCAGATGAGGCGTATAGAGAACGCGACAAGCAGGCAAGTG  211
            ||||||||||||||||||||||||||||||||||||||||||||||||||||||||||||
Sbjct  181  AAAATGGTGAGGGGGAAGACTCAGATGAGGCGTATAGAGAACGCGACAAGCAGGCAAGTG  240

Query  212  ACTTTTTCCAAGAGGAGAAATGGATTGTTGAAGAAGGCCTTCGAGCTTTCTGTTCTTTGT  271
            ||||||||||||||||||||||||||||||||||||||||||||||||||||||||||||
Sbjct  241  ACTTTTTCCAAGAGGAGAAATGGATTGTTGAAGAAGGCCTTCGAGCTTTCTGTTCTTTGT  300

Query  272  GATGCTGAAGTTGCTTTGATTATCTTCTCCCCTAGAGGCAAGCTCCATGAATTTGCCAGC  331
            ||||||||||||||||||||||||||||||||||||||||||||||||||||||||||||
Sbjct  301  GATGCTGAAGTTGCTTTGATTATCTTCTCCCCTAGAGGCAAGCTCCATGAATTTGCCAGC  360

Query  332  TCAAGCATGCATGAGACCATCGAACGATACCGCAAACACACTAAGGATGTTCAAAGTAAC  391
            ||||||||||||||||||||||||||||||||||||||||||||||||||||||||||||
Sbjct  361  TCAAGCATGCATGAGACCATCGAACGATACCGCAAACACACTAAGGATGTTCAAAGTAAC  420

Query  392  AACACGCCTGTGGCGCAAAACATGCAGCATCTAAAGCATGAAACAGCAACTCTGGCAAAG  451
            ||||||||||||| ||||||||||||||||||| ||||||||||||||||| ||||||||
Sbjct  421  AACACGCCTGTGGTGCAAAACATGCAGCATCTAGAGCATGAAACAGCAACTTTGGCAAAG  480

Query  452  AAGATAGAGCTTCTTGAAGTTTCAAAGAGGAAACTATTGGGAGAAGGTTTGGGATCGTGT  511
            ||||||||||||||||||||||||||||||||||||||||||||||||||||||||||||
Sbjct  481  AAGATAGAGCTTCTTGAAGTTTCAAAGAGGAAACTATTGGGAGAAGGTTTGGGATCGTGT  540

Query  512  TCCATTACTGAACTGCAACAGATAGAACAGCAGCTGGAGAAGAGTGTGAGCACTGTTCGA  571
            |||||||| |||||||||||||||||||||||||||||||||||||||||||||||||||
Sbjct  541  TCCATTACGGAACTGCAACAGATAGAACAGCAGCTGGAGAAGAGTGTGAGCACTGTTCGA  600

Query  572  GCCAGAAAGATGCAGGTCTTCAAAGAGCAGATCGAACAATTAAAGGAAAAGGAGAAGACT  631
            ||||||||||||||||||||||||||||||||||||||| |||| ||||||||||||| |
Sbjct  601  GCCAGAAAGATGCAGGTCTTCAAAGAGCAGATCGAACAACTAAAAGAAAAGGAGAAGAAT  660

Query  632  CTTGCAGCAGATAACGCAATGCTGCTTGCCAAGTATAAAGTTCAACCGCGACAAGAATCG  691
            |||||||||||||| ||||||||||||||||||||| |||||||||||||||||||||||
Sbjct  661  CTTGCAGCAGATAATGCAATGCTGCTTGCCAAGTATGAAGTTCAACCGCGACAAGAATCG  720

Query  692  CCAGAAGACGGAGGAAACATAACATCCACAACAGAAACCAGCGAGAACTCAGACGTGGAG  751
            ||||||||||||||||||||||||||||||||||||||||||||||||||||||||||||
Sbjct  721  CCAGAAGACGGAGGAAACATAACATCCACAACAGAAACCAGCGAGAACTCAGACGTGGAG  780

Query  752  ACTGAGCTGTTCATCGGACCTCCAGAAAAGAGATTCAAGCGCATTATGGAGAAATGATAC  811
            ||| ||||||||||||||||||||||||||||||||||||||||||||||||||||||||
Sbjct  781  ACTAAGCTGTTCATCGGACCTCCAGAAAAGAGATTCAAGCGCATTATGGAGAAATGATAC  840

Query  812  TATGCATGCACGTGGCTTGTTGCTTTTGATTTCGCTTGTCTAGTTCTTTACATAGTTGTC  871
            ||||||||||||||||||||||||||||||||||||||||||||||||||||||||||||
Sbjct  841  TATGCATGCACGTGGCTTGTTGCTTTTGATTTCGCTTGTCTAGTTCTTTACATAGTTGTC  900

Query  872  CAAGAAAAAACTCTTGTTTCTCATCAAACAATGACACAAATAATGGCTGGTTTGGTGAAT  931
            ||||||||||||||||||||||||||||||||||||||||||||||||||||||||||||
Sbjct  901  CAAGAAAAAACTCTTGTTTCTCATCAAACAATGACACAAATAATGGCTGGTTTGGTGAAT  960

Query  932  CATGTTTGTTGTTCCCTTGACACCAATGTTTGCA  965
            ||||||||||||||||||||||
Sbjct  961  CATGTTTGTTGTTCCCTTGACA------------  982
```

Fig. S5 Comparison of mRNA sequences of *DcSOC1-1* and *DcSOC1-2* in the reference carrot genome of DH1. Single nucleotide polymorphisms (SNPs) and insertion/deletions (InDels) are indicated with red boxes.

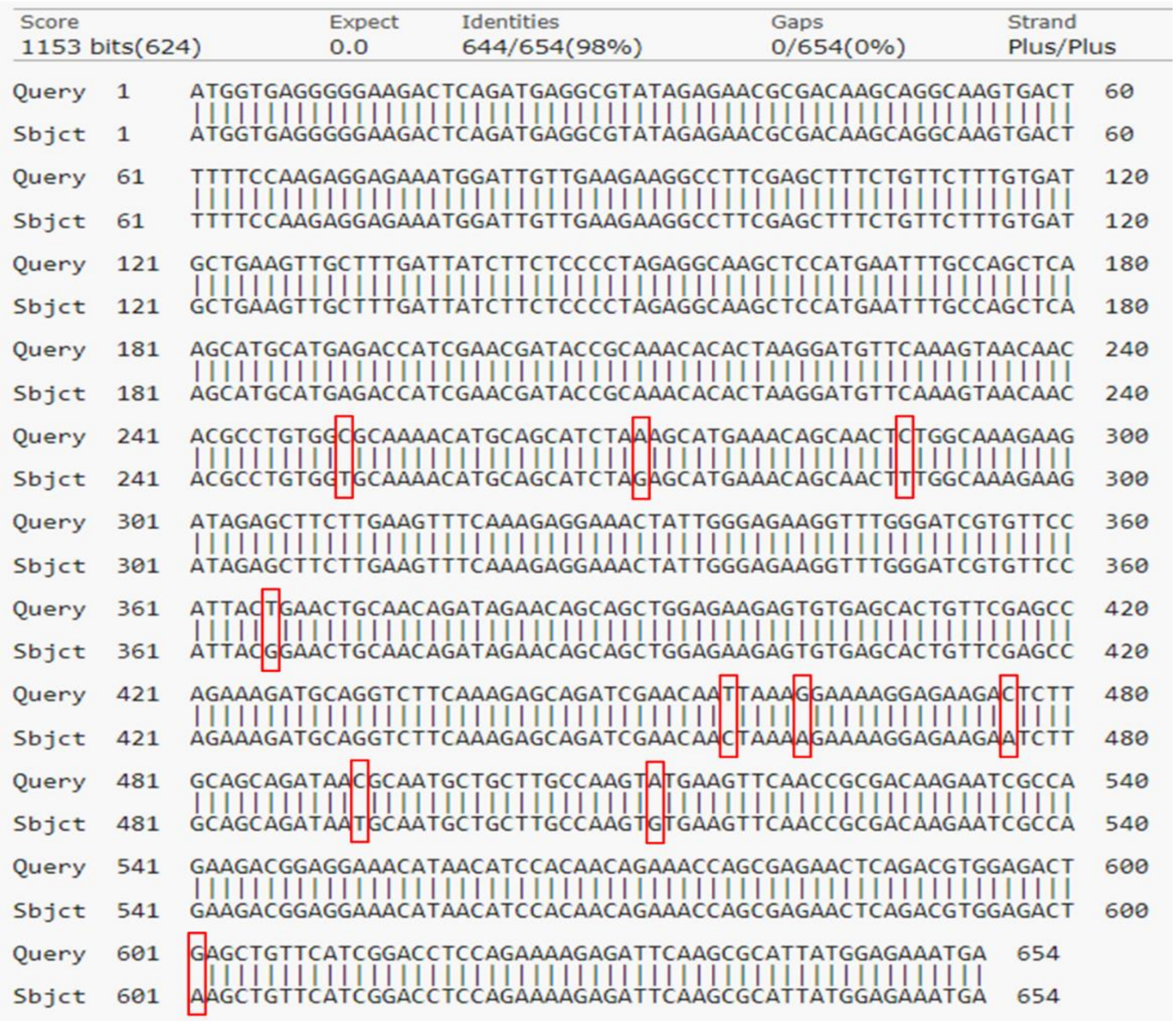

Fig. S6 Comparison of the coding sequences of *DcSOC1-1* (Query; LOC108209100) and *DcSOC1-2* (Sbjct; LOC108209496) in the reference carrot genome of DH1. SNPs differentiating both CDS are indicated with red boxes. The identity percentage between both CDS is 98%.

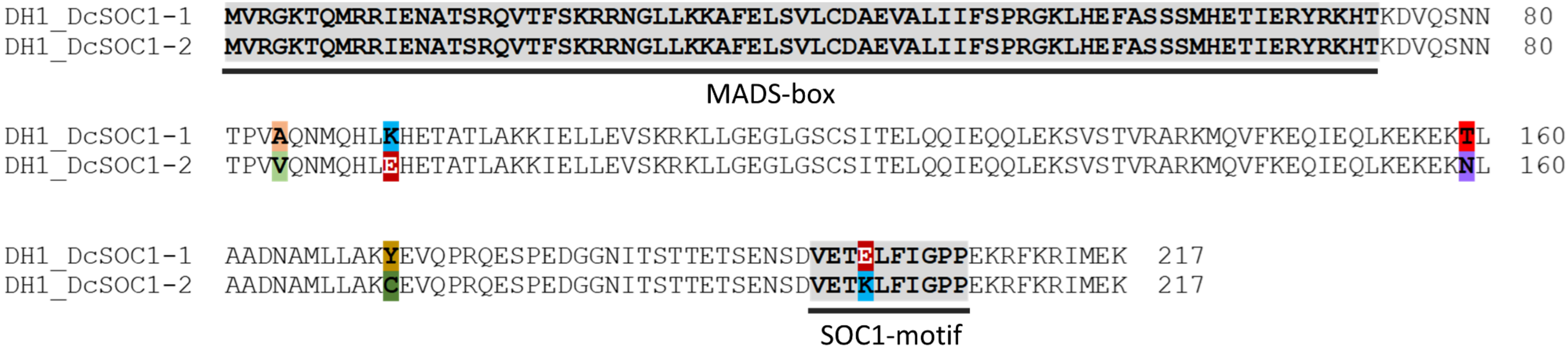

Fig. S7 Comparison of the predicted protein sequences of *DcSOC1-1* and *DcSOC1-2* from the carrot reference genome of DH1. The conserved MADS-box domain and SOC1-specific motif are indicated with underlines, and amino acid substitutions are highlighted in color. The identity percentage between both proteins is 98%.

```
B5-DcSOC1-1         TGCAAACATTGGTGTCAAGGGAACAACAAACATGATTCACCAAACCAGCCATTATTTGTGTCATTGTTTGATGAGAAACAAGAGTTTTTTCTTGGACAACTATGTAAAGAACTAGACAAG 120
Criolla-DcSOC1-1    TGCAAACATTGGTGTCAAGGGAACAACAAACATGATTCACCAAACCAGCCATTATTTGTGTCATTGTTTGATGAGAAACAAGAGTTTTTTCTTGGACAACTATGTAAAGAACTAGACAAG 120
B5-DcSOC1-2         TGCAAACATTGGTGTCAAGGGAACAACAAACATGATTCACCAAACCAGCCATTATTTGTGTCATTGTTTGATGAGAAACAAGTACTTTTTCTTGGACAACTATGTAAAGAACTAGACAAG 120
Criolla-DcSOC1-2    TGCAAACATTGGTGTCAAGGGAACAACAAACATGATTCACCAAACCAGCCATTATTTGTGTCATTGTTTGATGAGAAACAAGAGTTTTTTCTTGGACAACTATGTAAAGAACTAGACAAG 120
                    **********************************************************************************   ***********************************

B5-DcSOC1-1         CAGAATCAAAAGCAACAAGCCACGTGCATGCATAGTATCATTTCTCCATAATGCGCTTGAATCTCTTTTCTGGAGGTCCGATGAACAGTTCAGTCTCCACGTCTGAGTTCTCGCTGGTTT 240
Criolla-DcSOC1-1    CGAAATCAAAAGCAACAAGCCACGTGCATGCATAGTATCATTTCTCCATAATGCGCTTGAATCTCTTTTCTGGAGGTCCGATGAACAGCTCAGTCTCCACGTCTGAGTTCTCGCTGGTTT 240
B5-DcSOC1-2         CGAAATCAAAAGCAACAAGCCACGTGCATGCATAGTATCATTTCTCCATAATGCGCTTGAATCTCTTTTCTGGAGGTCCGATGAACAGCTTAGTCTCCACGTCTGAGTTCTCGCTGGTTT 240
Criolla-DcSOC1-2    CGAAATCAAAAGCAACAAGCCACGTGCATGCATAGTATCATTTCTCCATAATGCGCTTGAATCTCTTTTCTGGAGGTCCGATGAACAGCTTAGTCTCCACGTCTGAGTTCTCGCTGGTTT 240
                    *  ************************************************************************************* * *****************************

B5-DcSOC1-1         CTGTTGTGGATGTTATGTTTCCTCCGTCTTCTGGCGATTCTTGTCGCGGTTGAACTTCATACTTGGCAAGCAGCATTGCGTTATCTGCTGCAAGAGTCTTCTCCTTTTCCTTTAATTGTT 360
Criolla-DcSOC1-1    CTGTTGTGGATGTTATGTTTCCTCCGTCTTCTGGCGATTCTTGTCGCGGTTGAACTTCATACTTGGCAAGCAGCATTGCGTTATCTGCTGCAAGAGTCTTCTCCTTTTCCTTTAATTGTT 360
B5-DcSOC1-2         CTGTGGTGGATGTTATGTTTCCTCCGTCTTCTGGCGATTCTTGTCGCGGTTGAACTTCACACTTGGCAAGCAGCATTGCATTATCTGCTGCAAGATTCTTCTCCTTTTCTTTTAGTTGTT 360
Criolla-DcSOC1-2    CTGTTGTGGATGTTATGTTTCCTCCGTCTTCTGGCGATTCTTGTCGCGGTTGAACTTCACACTTGGCAAGCAGCATTGCATTATCTGCTGCAAGATTCTTCTCCTTTTCTTTTAGTTGTT 360
                    **** ************************************************************************** *************** ************* **** *****

B5-DcSOC1-1         CGATCTGCTCTTTGAAGACCTGCATCTTTCTGGCTCGAACAGTGCTCACACTCTTCTCCAGCTGCTGTTCTATCTGTTGCAGTTCAGTAATGGAACACGATCCCAAACCTTCTCCCAATA 480
Criolla-DcSOC1-1    CGATCTGCTCTTTGAAGACCTGCATCTTTCTGGCTCGAACAGTGCTCACACTCTTCTCCAGCTGCTGTTCTATCTGTTGCAGTTCAGTAATGGAACACGATCCCAAACCTTCTCCCAATA 480
B5-DcSOC1-2         CGATCTGCTCTTTGAAGACCTGCATCTTTCTGGCTCGAACAGTGCTCACACTCTTCTCCAGCTGCTGTTCTATCTGTTGCAGTTCCGTAATGGAACACGATCCCAAACCTTCTCCCAATA 480
Criolla-DcSOC1-2    CGATCTGCTCTTTGAAGACCTGCATCTTTCTGGCTCGAACAGTGCTCACACTCTTCTCCAGCTGCTGTTCTATCTGTTGCAGTTCCGTAATGGAACACGATCCCAAACCTTCTCCCAATA 480
                    ************************************************************************************* **********************************

B5-DcSOC1-1         GTTTCCTCTTTGAAACTTCAAGAAGCTCTATCTTCTTTGCCAGAGTTGCTGTTTCATGCTTTAGATGCTGCATGTTTTGCGCCACAGGCGTGTTGTTACTTTGAACATCCTTAGTGTGTT 600
Criolla-DcSOC1-1    GTTTCCTCTTTGAAACTTCAAGAAGCTCTATCTTCTTTGCCAGAGTTGCTGTTTCATGCTTTAGATGCTGCATGTTTTGCGCCACAGGCGTGTTGTTACTTTGAACATCCTTAGTGTGTT 600
B5-DcSOC1-2         GTTTCCTCTTTGAAACTTCAAGAAGCTCTATCTTCTTTGCCAAAGTTGCTGTTTCATGCTTTAGATGCTGCATGTTTTGCACCACAGGCGTGTTGTTACTTTGAACATCCTTAGTGTGTT 600
Criolla-DcSOC1-2    GTTTCCTCTTTGAAACTTCAAGAAGCTCTATCTTCTTTGCCAAAGTTGCTGTTTCATGCTCTAGATGCTGCATGTTTTGCACCACAGGCGTGTTGTTACTTTGAACATCCTTAGTGTGTT 600
                    ****************************************** ***************** ******************* ***************************************

B5-DcSOC1-1         TGCGGTATCGTTCGATGGTCTCATGCATGCTTGAGCTGGCAAATTCATGGAGCTTGCCTCTAGGGGAGAAGATAATCAAAGCAACTTCAGCATCACAAAGAACAGAAAGCTCGAAGGCCT 720
Criolla-DcSOC1-1    TGCGGTATCGTTCGATGGTCTCATGCATGCTTGAGCTGGCAAATTCATGGAGCTTGCCTCTAGGGGAGAAGATAATCAAAGCAACTTCAGCATCACAAAGAACAGAAAGCTCGAAGGCCT 720
B5-DcSOC1-2         TGCGGTATCGCTCGATGGTCTCATGCATGCTTGAGCTGGCAAATTCATGGAGCTTGCCTCTAGGGGAGAAGATAATCAAAGCAACTTCAGCATCACAAAGAACAGAAAGCTCGAAGGCCT 720
Criolla-DcSOC1-2    TGCGGTATCGTTCGATGGTCTCATGCATGCTTGAGCTGGCAAATTCATGGAGCTTGCCTCTAGGGGAGAAGATAATCAAAGCAACTTCAGCATCACAAAGAACAGAAAGCTCGAAGGCCT 720
                    ********** *************************************************************************************************************

B5-DcSOC1-1         TCTTCAACAATCCATTTCTCCTCTTGGAAAAAGTCACTTGCCTGCTTGTCGCGTTCTCTATACGCCTCATCTGAGTCTTCCCCCTCACCATTTTCGATCTCCACAACAGAAGAGGAAATG 840
Criolla-DcSOC1-1    TCTTCAACAATCCATTTCTCCTCTTGGAAAAAGTCACTTGCCTGCTTGTCGCGTTCTCTATACGCCTCATCTGAGTCTTCCCCCTCACCATTTTCGATCTCCACAACAGAAGAGGAAATG 840
B5-DcSOC1-2         TCTTCAACAATCCATTTCTCCTCTTGGAAAAAGTCACTTGCCTGCTTGTCGCGTTCTCTATACGCCTCATCTGAGTCTTCCCCCTCACCATTTTCGATCTCCACAACAGAAGAGGAAATG 840
Criolla-DcSOC1-2    TCTTCAACAATCCATTTCTCCTCTTGGAAAAAGTCACTTGCCTGCTTGTCGCGTTCTCTATACGCCTCATCTGAGTCTTCCCCCTCACCATTTTCGATCTCCACAACAGAAGAGGAAATG 840
                    ************************************************************************************************************************

B5-DcSOC1-1         GAAAAAGAAACAAGAGAAGAGATGATTTAGGTGGAGGAAAGATAGGCCTAAAAAAGGCAAAGAAACAACTTTATAAAGGGGAAGAGAGATCCCAAATTATTTCCAAGAGAGCACAGAACA 960
Criolla-DcSOC1-1    GAAAAAGAAACAAGAGAAGAGATGATTTAGGTGGAGGAAAGATAGGCCTAAAAAAGGCAAAGAAACAACTTTATAAAGGGGAAGAGAGATCCCAAATTATTTCCAAGAGAGCACAGAACA 960
B5-DcSOC1-2         GAAAAAGAAACAAGAGAAGAGATGATTTAGGTGGAGGAAAGATAGGCCTAAAAAAGGCAAAGAAACAACTTTATAAAGGGGAAGAGAGATCCCAAATTATTTCCAAGAGAGCACAGAACA 960
Criolla-DcSOC1-2    GAAAAAGAAACAAGAGAAGAGATGATTTAGGTGGAGGAAAGATAGGCCTAAAAAAGGCAAAGAAACAACTTTATAAAGGGGAAGAGAGATCCCAAATTATTTCCAAGAGAGCACAGAACA 960
                    ************************************************************************************************************************

B5-DcSOC1-1         AGCCC 965
Criolla-DcSOC1-1    AGCCC 965
B5-DcSOC1-2         AGCCC 965
Criolla-DcSOC1-2    AGCCC 965
                    *****
```

Fig. S8 Alignment of mRNA sequences of *DcSOC1-1* and *DcSOC1-2* from B5 and Criolla INTA. Nucleotide polymorphisms are indicated in color.

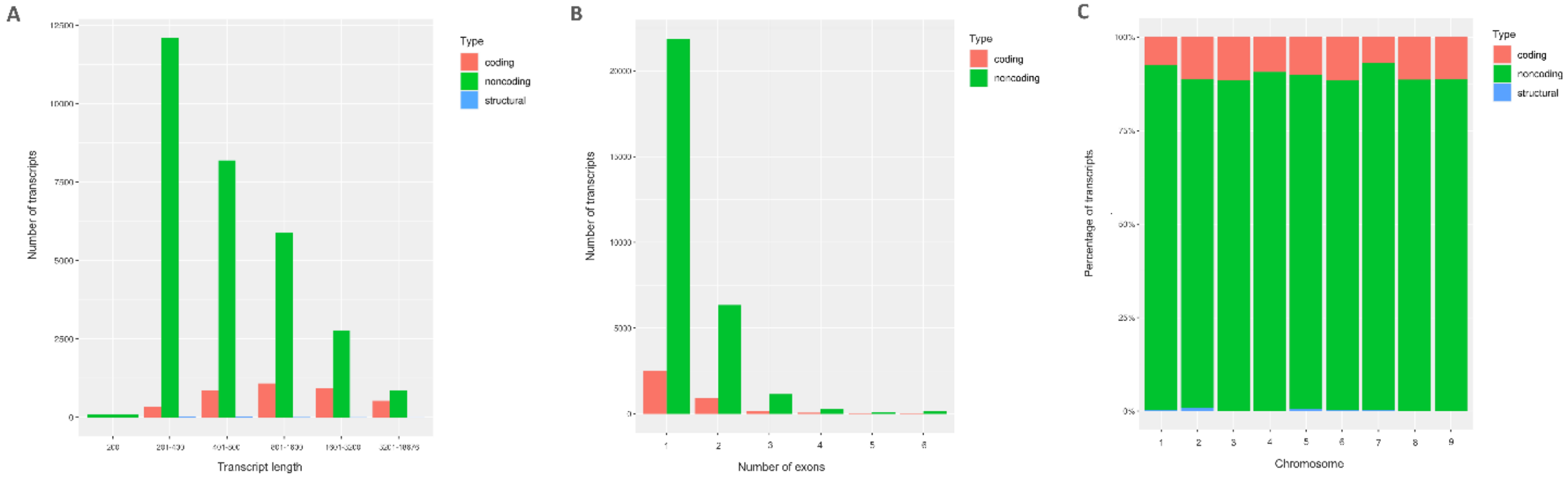

Fig. S9 Characterization of new carrot transcripts from apical meristems. (**A**) Transcript length distribution for the new coding, noncoding, and structural RNAs. (**B**) Number of exons per transcript for the new coding and noncoding RNAs. (**C**) Percentual distribution of the new coding, noncoding, and structural RNAs along the chromosomes.
